# mRNA recognition and packaging by the human transcription-export complex

**DOI:** 10.1038/s41586-023-05904-0

**Published:** 2023-04-05

**Authors:** Belén Pacheco-Fiallos, Matthias K. Vorländer, Daria Riabov-Bassat, Laura Fin, Francis J. O’Reilly, Farja I. Ayala, Ulla Schellhaas, Juri Rappsilber, Clemens Plaschka

**Affiliations:** 1 Research Institute of Molecular Pathology (IMP), Vienna BioCenter (VBC), Campus Vienna-Biocenter 1, 1030, Vienna, Austria; 2 Vienna BioCenter PhD Program, Doctoral School of the University of Vienna and Medical University of Vienna, 1030, Vienna, Austria; 3 Bioanalytics Unit, Institute of Biotechnology, Technische Universität Berlin, 13355 Berlin, Germany; 4 Wellcome Centre for Cell Biology, University of Edinburgh, Max Born Crescent, Edinburgh, EH9 3BF, UK

## Abstract

Newly made messenger RNAs are processed and packaged into ribonucleoprotein complexes (mRNPs) and recognized by the essential transcription-export complex (TREX) for nuclear export^1,2^. However, the mechanisms of mRNP recognition and three-dimensional organization are poorly understood^3^. Here, we report cryo-electron microscopy and tomography structures of reconstituted and endogenous human mRNPs bound to the two-megadalton TREX complex. We show that mRNPs are recognized through multivalent interactions between the TREX subunit ALYREF and mRNP-bound exon-junction complexes. Exon-junction complexes can multimerize through ALYREF, suggesting a mechanism for mRNP organization. Endogenous mRNPs form compact ‘globules’ that are coated by multiple TREX complexes. These results reveal how TREX may simultaneously recognize, compact, and protect mRNAs to promote their packaging for nuclear export. The mRNP globule organization provides a framework to understand how mRNP architecture could facilitate mRNA biogenesis and export.

## Introduction

Eukaryotic gene expression requires the selective transport of mature mRNAs from the nucleus to the cytoplasm for translation. In the nucleus, mRNAs are capped, spliced, cleaved, poly-adenylated and bound by various proteins to form mature ribonucleoprotein complexes (mRNPs)^1–5^. These spliced mRNPs contain the cap-binding complex (CBC) at the mRNA 5’-end, the exon junction complex (EJC) upstream of every splice junction, and the poly(A)-binding protein at the mRNA 3’-end^1,2^. The mRNPs are then recognized by the conserved and essential transcription-export complex (TREX), which requires its DExD-box ATPase subunit UAP56 (yeast Sub2) and the mRNA export adapter ALYREF (yeast Yra1)^1,4-8^. TREX subsequently licenses loading of the mRNA export factor, NXF1—NXT1, onto these mRNPs, which enables their nuclear export^9–12^. During this process, TREX discriminates mRNAs from their immature precursors and other nuclear RNAs^13^. Additionally, TREX prevents the formation of RNA-DNA hybrids and thereby protects genome integrity^14^. How TREX selectively recognizes and subsequently acts on mRNPs is poorly understood.

Data on a select number of nuclear mRNPs suggest that these are organized and compacted to facilitate mRNA transport and export^15,16^. Yet unlike the higher-order organization of DNA^17^, how human mRNAs are generally organized in three-dimensions and packaged remains poorly understood^2,3,15,18^. The EJC has been implicated in this process^2,18^, which has on average eight binding sites (splice junctions) per human mRNA^19^, and is thus a major mRNP component^20^. mRNA-bound EJCs were suggested to interact with each other through an unknown mechanism, which can organize mRNPs^18,21^. The TREX subunit ALYREF binds to the EJC^1–4^, suggesting that TREX-mediated recognition of mRNPs and their organization could be linked. However, a mechanistic and structural framework for general mRNP organization is lacking.

Here, we reconstituted human TREX core–mRNP complexes from recombinant TREX and EJC proteins *in vitro* and purified endogenous TREX–mRNPs from human cells. We integrated cryo-EM, cryo-tomography, protein crosslinking and biochemistry to resolve their structures and show that TREX binds mRNA-bound EJCs through multivalent interactions with ALYREF. This can explain how spliced mRNPs are recognized and organized. We further show that human mRNPs form compact globules that are coated with TREX complexes. Our results reveal the architectures of native mRNPs and suggest mechanisms of nuclear mRNA recognition and packaging.

## Results And Discussion

### Cryo-EM of ALYREF–exon-junction complexes

**Extended Data Figure 1:**
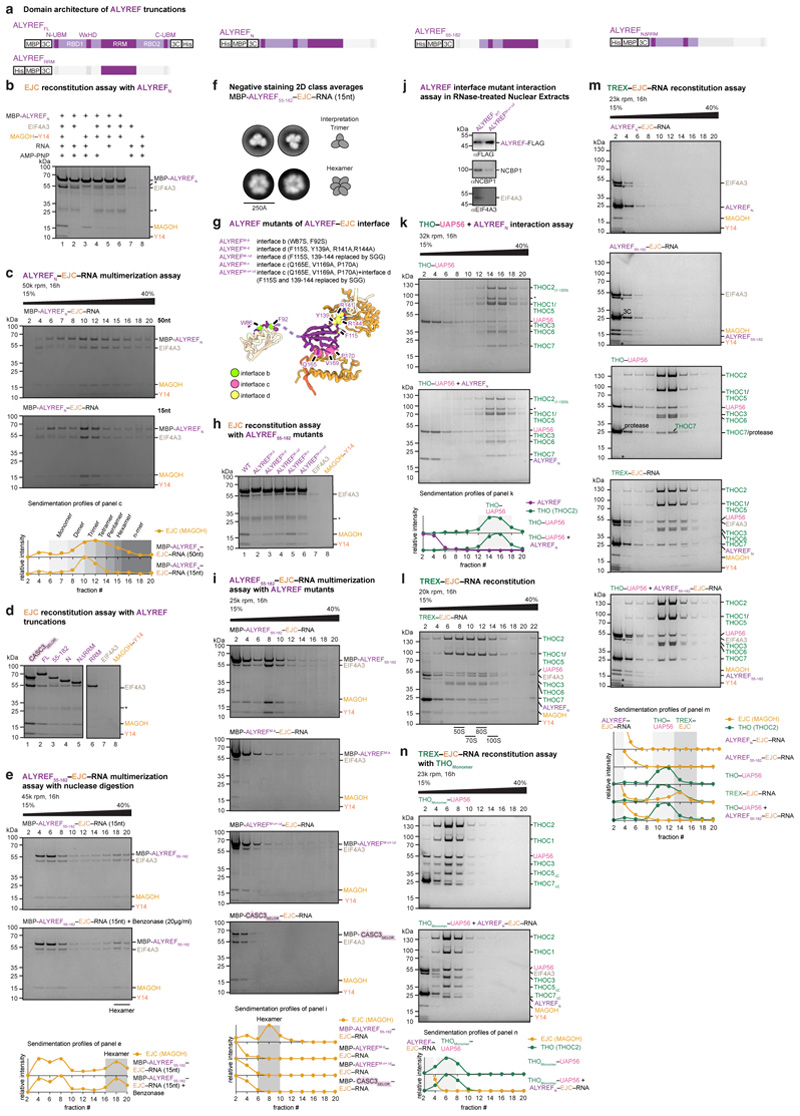
Biochemical characterization of TREX–EJC–RNA and ALYREF–EJC–RNA complexes. **a.** Domain architecture of ALYREF constructs and their nomenclature used throughout. N- and C-UBM, N- and C-terminal UAP56-binding motif; RBD1 and RBD2, RNA-binding domain 1 and 2; RRM, RNA-recognition motif; MBP, Maltose Binding Protein; 3C, PreScission protease cleavage site; His, Histidine-tag. **b.** ALYREF_N_ reconstitutes the EJC *in vitro*. Pulldown assay with MBP-ALYREF_N_ (bait) incubated with EIF4A3, MAGOH–Y14 (residues 66-154), or both, with or without a 15 nucleotide long single stranded (ss) RNAs and/or AMP-PNP. Complex formation was determined by SDS-PAGE analysis with Coomassie blue staining. This exact experiment was done once, but similar results were obtained in two additional experiments either without AMP-PNP or without RNA. **c.** ALYREF_N_–EJC–RNA complexes form multimers. ALYREF_N_–EJC–RNA was assembled on 50 (top) or 15 nucleotides (nt) long single stranded RNAs (ssRNAs) (bottom) and analyzed in sucrose density gradients. SDS-PAGE analysis with Coomassie blue staining of gradient fractions indicates multiple oligomeric states. The sucrose gradient sedimentation profile (bottom) is based on quantification of MAGOH band intensities. The sedimentation coefficients were estimated in *CowSuite* based on the predicted molecular weights of the different oligomeric states (an ALYREF_N_–EJC–RNA monomer is ~150 kDa). The sedimentation range of one to six ALYREF_N_–EJC–RNA complexes is indicated. We analyzed even fraction numbers and included fractions 7 and 15 to better resolve monomer and hexamer peaks using SDS-PAGE. Gradient conditions are specified on top. This exact experiment was done once, but ALYREF_N_–EJC–RNA multimerization was similarly observed in an experiment with different gradient ultracentrifugation parameters. **d.** The ALYREF WxHD domain is sufficient for EJC reconstitution. Pulldown assay with different MBP-ALYREF truncation constructs (see panel **a**) or MBP-CASC3_SELOR_ as a bait and EIF4A3 and MAGOH–Y14 (residues 66-154) to probe EJC-reconstitution efficiency. Complex formation was determined by SDS-PAGE analysis with Coomassie blue staining. This experiment was done twice. For gel source data, see Supplementary Figure 3. **e.** ALYREF55-182–EJC–RNA oligomers form *in vitro*, are resistant to RNase treatment, and do not require the ALYREF RED1 and UBM domains. The ALYREF_55-182_–EJC– RNA complex was assembled on 15 nt ssRNA and treated (bottom) or not treated (top) with 20 μg benzonase mL^-1^ to digest protein-unbound RNA. The complexes were then analyzed in sucrose density gradients. SDS-PAGE analysis with Coomassie blue staining of gradient fractions indicates indistinguishable oligomeric sedimentation profiles of the ALYREF_55-182_–EJC–RNA complex, with or without benzonase digestion. The sucrose gradient sedimentation profile (bottom) is based on quantification of MAGOH band intensities. The hexamer peak (confirmed by negative staining, see panel **f**) is indicated with a grey box. Gradient conditions are specified. This experiment was done twice, the second time with 2 μg benzonase mL^-1^. **f.** Negative stain 2D class averages show that MBP-ALYREF_55-182_–EJC–RNA (15nt) complexes form trimeric (top) and hexameric (bottom) complexes. Cartoon interpretations are shown on the right. Scale bar, 250 Å. **g.** Ribbon model showing the location of mutated residues in ALYREF in the ALYREF-EJC interface. Mutated residues are shown as sticks and Cα-spheres colored by the ALYREF–EJC interface. **h.** ALYREF–EJC interface mutations in the ALYREF55-182 constructs reduce the efficiency of EJC reconstitution. The pulldown assay was carried out as in panel **d**. Mutated residues are indicated in panel **g**. Complex formation was determined by SDS-PAGE analysis with Coomassie blue staining. This experiment was done twice. **i.** Mutation of ALYREF in the ALYREF–EJC interfaces impairs ALYREF–EJC–RNA complex oligomerization *in vitro*. ALYREF^M-*b*^ and ALYREF^M-*c*+Δ*d*^ mutants were made in the ALYREF55-182 construct (see panel **g** for mutant details). Wild-type or mutant ALYREF55-182 or the isolated CASC3_SELOR_ were used to assemble EJC–RNA complexes on a 15 nt long RNA and analyzed in sucrose density gradients for their multimerization. SDS-PAGE analysis with Coomassie blue staining of gradient fractions indicates loss of high-order oligomers in the sedimentation profiles of ALYREF mutants, which resemble the pattern of the monomeric CASC3_SELOR_. The sucrose gradient sedimentation profile (bottom) is based on quantification of MAGOH band intensities. The hexamer peak is indicated with a grey rectangle. Gradient conditions are specified on top. This exact experiment was done once. **j.** The *in vivo* mutation of ALYREF in the ALYREF–EJC interface (mutant ALYREF^M-*c*+Δ d^; see panel **g** for details) impairs its interaction with mRNP components. Wild-type FLAG-tagged ALYREF_WT_ or the FLAG-ALYREF^M-*c*+Δ d^ mutant were ectopically overexpressed in K562 cells, which also ectopically overexpressed THOC1-GFP. The two cell lines were used to prepare nuclear extract (NE), which were then treated with benzonase for 16 h at 4°C, including a final concentration of 5 mM MgCl_2_. The benzonase-treated extracts were then applied to anti-FLAG M2 resin for purification. Western blot analysis shows wild-type ALYREF or the mutant ALYREF^M-*c*+Δ d^ (via their FLAG-tag), NCBP1, and EIF4A3. This experiment was done twice. **k.** The THO–UAP56 complex does not form a complex with ALYREF_N_ in sucrose density gradients, suggesting that UAP56 binds the ALYREF UBM with low affinity as observed in yeast^31^. SDS-PAGE stained with Coomassie blue. The sucrose gradient sedimentation profile (bottom) is based on quantification of THOC2 and ALYREF band intensities. Gradient conditions are specified on top. This experiment was done twice. For gel source data, see Supplementary Figure 4. **l.** *In vitro* reconstitution of TREX–EJC–RNA. The recombinant proteins or subcomplexes were mixed as shown in Fig. 1d and applied to sucrose density gradient ultracentrifugation. SDS-PAGE analysis with Coomassie blue staining confirms the formation of a complex containing all eleven proteins subunits and a sedimentation coefficient of ~75 S. Gradient conditions are specified on top. This experiment was done four times. **m.** The ALYREF-UBM–UAP56 interaction is required to form the TREX–EJC–RNA complex *in vitro*. Sucrose gradient sedimentation profiles of (from top to bottom): ALYREF_N_–EJC–RNA, ALYREF55-182–EJC–RNA, THO–UAP56, THO–UAP56 with ALYREF_N_–EJC–RNA, and THO–UAP56 with ALYREF55-182–EJC–RNA. Gradient fractions were analyzed by SDS-PAGE and Coomassie staining. Bellow, sucrose gradient sedimentation profiles are based on quantifications of the EJC subunit MAGOH and THO complex subunit THOC2 band intensities. MAGOH intensities were multiplied by a factor of 3 for better visualization. Gradient fractions containing ALYREF–EJC–RNA (light grey), THO–UAP56 (light grey), or TREX–EJC–RNA (grey) are shown with rectangles. Gradient conditions are specified on top. This experiment was done five times. **n.** A monomeric THO complex (THO_Monomer_) does not form TREX–EJC–RNA complexes *in vitro*. Sucrose gradient sedimentation profiles of THO_Monomer_–UAP56 (see Methods for details) alone or in presence of ALYREF_N_–EJC–RNA, assembled on a 15nt ssRNA. Gradient fractions were analyzed by SDS-PAGE and Coomassie staining. THO_Monomer_–UAP56 did not form TREX–EJC–RNA complexes (compare to panel **m**). Below, sucrose gradient sedimentation profiles are based on quantifications of the EJC subunit MAGOH and THO complex subunit THOC2 band intensities. MAGOH intensities were multiplied by a factor of 3 for better visualization. Gradient conditions are specified on top. This experiment was done twice.

**Extended Data Figure 2:**
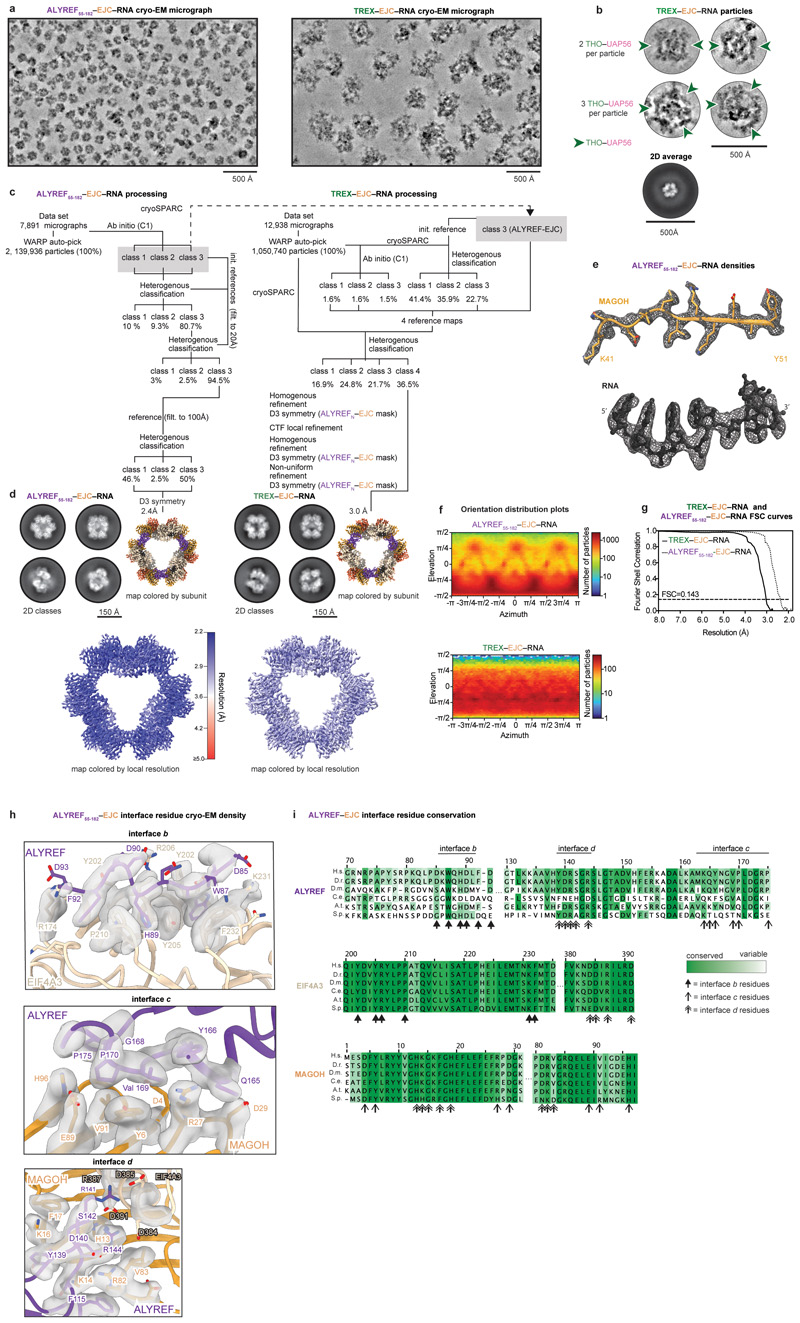
ALYREF–EJC–RNA and TREX–EJC–RNA complex cryo-EM image processing and structural details. **a.** Denoised cryo-EM micrographs of ALYREF_55-182_–EJC–RNA (left) and TREX–EJC– RNA (right) complexes (see Methods). Scale bar, 500 Å. The ALYREF_55-182_–EJC–RNA dataset contained 7,891 micrographs and the TREX–EJC–RNA dataset 12,938 micrographs, respectively. **b.** TREX–EJC–RNA complexes contain multiple THO–UAP56 complexes, caging in a central ALYREF_N_–EJC–RNA complex. Single TREX–EJC–RNA particles from a denoised cryo-EM micrograph can contain two (left) or three (right) THO–UAP56 complexes. In 2D class averages, the THO–UAP56 complexes blur out, because the central ALYREF_N_–EJC–RNA complex is aligned (bottom). **c.** Three-dimensional image classification tree of ALYREF_55-182_–EJC–RNA (left) and TREX–EJC–RNA (right) cryo-EM data. The ALYREF_55-182_–EJC–RNA dataset contained 7,891 micrographs from which 2,139,936 particles were picked and extracted. Three initial volumes were generated from 100,000 particles in cryoSPARC^64^ using the *ab-initio* reconstruction algorithm, which served as reference volumes to classify the entire dataset using three rounds of heterogenous classification (*see* Methods). The final particle stack contained 1,564,602 particles and was refined to 2.4 Å using D3 symmetry. The TREX–EJC–RNA dataset contained 1,050,740 particles, which were classified using initial volumes obtained from the ALYREF_55-182_–EJC–RNA dataset and from *ab initio* reconstructions. After 3D classification, 3D refinement and application of D3 symmetry in cryoSPARC^64^ yielded a 3.0 Å resolution map from 383,520 particles. The type of mask is indicated for each 3D refinement. Please refer to Methods for further details. **d.** ALYREF_55-182_–EJC–RNA and TREX–EJC–RNA give rise to indistinguishable 2D classes and reconstructions (top left and right), apart from the higher resolution of the ALYREF_55-182_–EJC–RNA dataset (bottom left), which contains more particles. **e.** Representative protein (MAGOH, top) and RNA (bottom) densities from the 2.4 Å resolution ALYREF_55-182_–EJC–RNA map. **f.** Orientation distribution plots for all particles contributing to the ALYREF_55-182_–EJC– RNA and TREX–EJC–RNA cryo-EM map, visualized in cryoSPARC^64^. **g.** Gold-standard Fourier shell correlation (FSC = 0.143) of the ALYREF_55-182_–EJC–RNA and TREX–EJC–RNA cryo-EM maps. **h.** Cryo-EM densities for ALYREF–EJC interface residues from the 2.4 Å ALYREF_55-182_–EJC–RNA map. **i.** Multiple sequence alignment showing the conservation of ALYREF–EJC interface residues in ALYREF, EIF4A3, and MAGOH using human (H.s.), *Danio rerio* (D.r.), *Drosophila melanogaster* (D.m.), *Caenorhabditis elegans* (C.e.), *Arabidopsis thaliana* (A.t.), and *Schizosaccharomycespombe* (S.p) sequences. A different type of arrow is used to indicate residues of interfaces *b, c*, and *d*.

**Figure 1:**
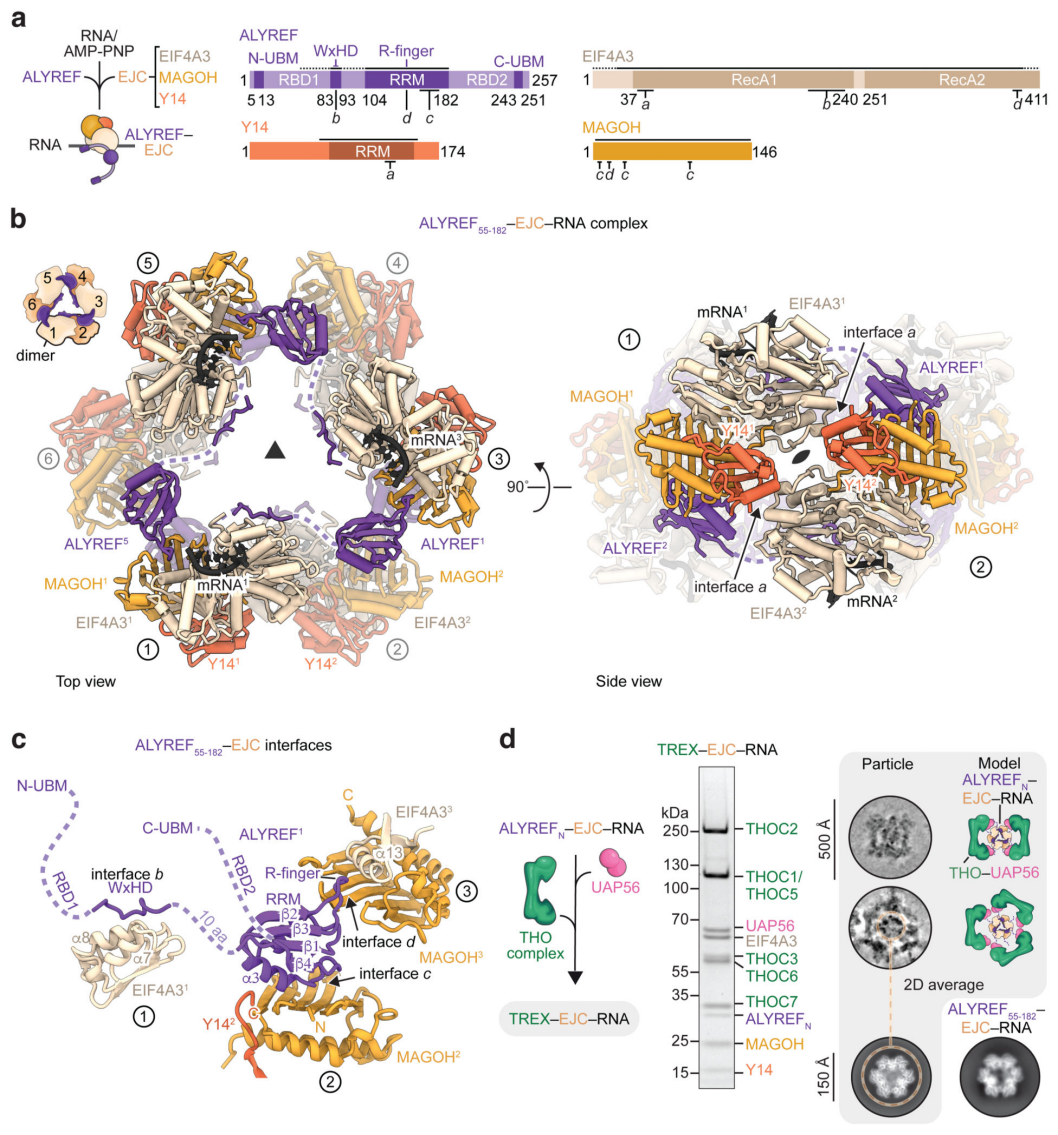
Structure of an ALYREF-exon-junction complex oligomer. **a.** Assembly scheme (left) and domain organization (right) of ALYREF–EJC–RNA complex components (see Extended Data Fig. 1 and Methods for details). Solid lines indicate regions included in the atomic model, dotted lines indicate protein construct boundaries. N- and C-terminal UAP56-binding motifs (N- and C-UBM); RNA-binding domains 1 and 2 (RBD1 and RBD2); RNA-recognition motif (RRM). The protein color code is used throughout. **b.** ALYREF_55-182_–EJC–RNA complex structure shown from top and side views. The structure shows an ALYREF_55-182_–EJC–RNA hexamer, one of the various oligomers observed *in vitro* (Extended Data Fig. 1c, f). In the top view (left), every second protomer is rendered transparent for clarity. In the side view (right) protomers 3 to 6 are transparent for clarity. In the cartoon inset ALYREF_55-182_–EJC–RNA protomers are labelled, 1 to 6, and the dimer is outlined in black. **c.** One ALYREF molecule bridges three EJCs, labelled 1, 2, and 3, through its WxHD (interface *b*) and RRM domains (interfaces *c* and *d*), suggesting mechanisms for mRNP recognition and packaging. The conserved ALYREF R144 arginine-finger (R-finger) of interface *d* wedges in between EIF4A3 and MAGOH of protomer 3. See Extended Data Fig. 2 for details. **d.** Assembly scheme (left), SDS-PAGE analysis (center), and cryo-EM (right) of the *in vitro* reconstituted TREX–EJC–RNA complex. Addition of recombinant THO–UAP56 to ALYREFN–EJC–RNA yields the TREX–EJC–RNA complex (center) (see also Extended Data Fig. 1l). Representative cryo-EM particles and their cartoon interpretations are shown on the right. Below, 2D averages of the ALYREF–EJC–RNA complex bound to THO–UAP56 or in isolation show an indistinguishable complex organization (see Extended Data Fig. 2d). THO complex (green), UAP56 (pink), all other protein colors as in panel **a**. For gel source data, see Supplementary Figure 1.

To understand how the TREX subunit ALYREF recognizes mRNPs and how mRNA-bound EJCs interact, we reconstituted the recombinant EJC, comprising EIF4A3, Y14, MAGOH, a 15-nucleotide RNA, and AMP-PNP, and a soluble ALYREF construct (ALYREF_N_, residues 1-182) (Fig. 1a, Extended Data Fig. 1a, b). ALYREF_N_ included the conserved N-terminal UAP56-binding motif (N-UBM), RNA-binding domain 1 (RBD1), WxHD, and RNA-recognition motif (RRM), but lacked the second RBD2 and C-terminal UBM (C-UBM) regions (Extended Data Fig. 1a). Reconstituted ALYREF_N_–EJC–RNA complexes formed a range of multimers independent of RNA length (15 or 50 nucleotides) (Extended Data Fig. 1c). We made ALYREF truncations and identified ALYREF(55-182) (residues 55-182) to be minimally required for efficient ALYREF–EJC–RNA reconstitution and multimerization (Extended Data Fig. 1d-f). Multimerization of ALYREF_55-182_–EJC–RNA complexes was resistant to nuclease treatment (Extended Data Fig. 1e), indicating that multimerization depends on protein-protein interfaces. To reveal the structural basis of ALYREF_55-182_–EJC interaction and multimerization, we purified an ALYREF_55-182_–EJC–RNA hexamer from among the various oligomers (Extended Data Fig. 1e, f), owing to its size, stability, and homogeneity, and subjected this complex to cryo-EM single particle analysis (Extended Data Fig. 2). Particle averaging yielded a 2.4 Å resolution density, enabling us to fit and extend crystallographic models of the EJC bound to six nucleotides of RNA^23^, the ALYREF RRM and the WxHD motif (Fig. 1b, Extended Data Fig. 2, Supplementary Text 1, Extended Data Table 1, Supplementary Video 1, Methods).

### ALYREF–exon-junction complex structure

In the structure, six copies of ALYREF(55-182)–EJC–RNA (protomers 1-6) join into a ring via multivalent and conserved ALYREF–EJC interactions (Extended Data Fig. 2h, i, Supplementary Text 1). Each protomer contributes one EJC–EJC and three ALYREF–EJC interfaces, which we label *a, b, c*, and *d* and describe for protomer 1 (Fig. 1b, c, Supplementary Video 2). The EJC–EJC interface *a* forms between the EJC subunit EIF4A3 N-terminus of protomer 1 and subunit Y14 of protomer 2 (Fig. 1b left). The ALYREF–EJC interface *b* contains the ALYREF WxHD element (protomers 1) that binds its own complex’s EIF4A3 (Fig. 1b, c)^23,24^ Interfaces *c* and *d* involve two surfaces on opposite sides of the ALYREF RRM. The RRM binds on one side to MAGOH of protomer 2 (interface *c*) and on the other inserts into an EIF4A3-MAGOH cavity in protomer 3 (interface *d*) through extended ALYREF loops and a conserved residue R144 that we name the ‘R-finger’. These three ALYREF–EJC interfaces (*b*, *c*, and *d*) reveal how ALYREF may specifically recognize spliced mRNPs and facilitate EJC multimerization^18,21^. In support of the structure, mutation of the ALYREF WxHD (interface *b*) or RRM domains (interfaces *c* and *d*) impair efficient *in vitro* ALYREF–EJC–RNA complex formation and ALYREF–EJC–RNA multimerization (Extended Data Fig. 1g-i). Further, mutation of the ALYREF WxHD or the RRM domains reduce ALYREF-mRNP binding in nuclear extracts (ref.^7^ and Extended Data Fig. 1j). Taken together, the data suggest that multivalent ALYREF–EJC interactions contribute to specific mRNP recognition.

In the structure, one molecule of ALYREF bridges three EJC–RNA complexes. We speculate that ALYREF may thereby use multivalent interactions to simultaneously recognize and organize mRNPs. This could be achieved by looping out the mRNA held between two EJC-bound splice junctions, which are brought into proximity by ALYREF, and thereby facilitate mRNP compaction (Supplementary Video 3). Further, the conserved ALYREF Arg-Gly-rich motifs in RBD1 and RBD2 domains are necessary to bind RNA *in vitro* (Extended Data Fig. 5k and ref.^25^) and yeast ALYREF can anneal RNA duplexes^26^. Thus, ALYREF, together with other multivalent RNA-binding proteins such as the Ser-Arg-rich SRSF family proteins, might also link nearby mRNA segments and further contribute to mRNP compaction^26^. Taken together, ALYREF- and EJC-mediated mRNP recognition and packaging^18,21^ may depend on multivalent proteinprotein and protein-mRNA interactions.

### ALYREF bridges mRNPs to THO–UAP56

We next investigated how the complete TREX complex recognizes a spliced mRNP and delivers the essential DExD-box ATPase UAP56^1,9,27^ to the mRNA. We built on our previous cryo-EM study of the recombinant human TREX core, THO-UAP56^28^, and added the ALYREF_N_–EJC–RNA complex (Fig. 1d). The 28-subunit TREX core comprised the tetrameric 24-subunit THO complex (THOC1, -2, -3, -5, -6, -7) and four copies of UAP56^7,28^. Fully formed TREX–EJC–RNA complexes sedimented at ~75S during sucrose gradient ultracentrifugation (Extended Data Fig. 1l) and were analyzed by cryo-EM (Extended Data Fig. 2). Notably, the imaged TREX–EJC–RNA particles contained ALYREF_N_–EJC–RNA at the center, caged by one to three flexibly linked THO–UAP56 complexes that blurred out during particle averaging (Fig. 1d, Extended Data Fig. 2a, b, Methods). Focused three-dimensional refinement of the ALYREF_N_–EJC–RNA complex yielded a 3.0 Å resolution density, which was indistinguishable from the density of the isolated 2.4 Å ALYREF(55-182)–EJC–RNA structure (Extended Data Fig. 2d). In the context of reconstituted TREX–EJC–RNA complexes, only the ALYREF WxHD and RRM regions contact EJC–RNA complexes, but not the N-UBM and RBD1 or other TREX subunits (Extended Data Fig. 2). Thus, ALYREF bridges the EJC–RNA complex to THO–UAP56 to form TREX–EJC–RNA complexes *in vitro*, likely through multiple copies of its conserved UAP56-binding motifs (UBMs)^29,30^. This may mirror how endogenous TREX assembles on mRNPs.

Biochemical data in nuclear extracts and *in vitro* support this model. First, endogenous ALYREF does not co-purify with the THO–UAP56 complex from heavily nuclease-digested K562 nuclear extract^28^, indicating that TREX assembly requires an mRNP substrate. Second, the previously reported yeast UAP56–ALYREF UBM interactions are of low affinity (K_D_ ~2.9 μΜ^31^), consistent with the lack of an *in vitro* interaction between recombinant human ALYREF_N_ and THO–UAP56 (Extended Data Fig. 1k). Third, TREX–EJC–RNA complexes do form *in vitro* with ALYREFN–EJC– RNA oligomers (Fig. 1d, Extended Data Fig. 1l, m), which can form multivalent ALYREF N-UBM to THO–UAP56 interactions. In addition, we show that multiple copies of the ALYREF_N_ UBM in the ALYREF_N_–EJC–RNA complex (Extended Data Fig. 1m) and a THO–UAP56 complex tetramer, but not a THO–UAP56 complex monomer, are jointly required for TREX–EJC–RNA complexes to form *in vitro* (Extended Data Fig. 1n). Fourth, mutation of the ALYREF RRM–EJC interfaces, which impairs ALYREF–EJC–RNA multimerization *in vitro* (Extended Data Fig. 1i), leads to loss of the ALYREF interaction with mRNP components in nuclear extract (Extended Data Fig. 1j). Taken together, the data show that multivalent interactions made by ALYREF and THO are likely important for TREX assembly on mRNPs and for efficient delivery of UAP56 to mRNAs.

**Extended Data Figure 3:**
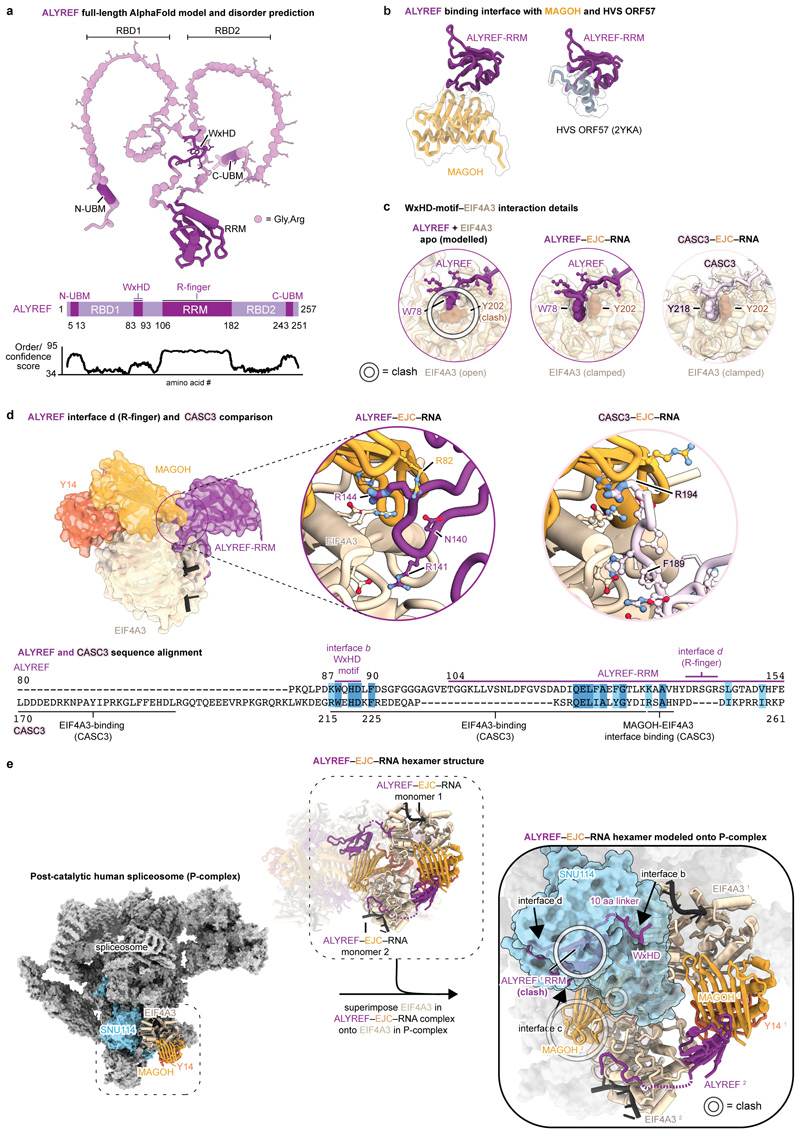
Comparisons of ALYREF–EJC interaction details with a viral ALYREF-ORF57 complex, the cytoplasmic CASC3–EJC–RNA complex, and the EJC-bound P-complex spliceosome. **a.** Organization of ALYREF. Top: Structural model of full-length ALYREF predicted with AlphaFold^62,63^. Annotated domains (N-UBM, WxHD motif, RRM and C-UBM) are colored in darker shades of purple. Spheres represent backbone atoms of glycine and arginine residues in the RBD domains. Middle: ALYREF domain diagram. Black bars indicate residues that are included as an atomic model in this study. Bottom: AlphaFold per residues confidence score (pLDDT) plot. High values are indicative of high confidence predictions, whereas low values represent residues that are likely disordered in solution. **b.** Comparison of the ALYREF RRM domain interaction with the EJC subunit MAGOH (interface *c*, left) and the Herpes simplex virus ORF57 (right)^34^. ALYREF binds viral ORF57 differently compared to the overlapping ALYREF–EJC interface *c*. This supports a general model that ALYREF can use multiple interfaces to engage either viral proteins, such as ORF57, or mRNP maturation marks, such as the CBC or EJCs, and may enable ALYREF to broadly select its RNA targets. **c.** Details of the WxHD motifs binding to the EJC. Left: Modelling of apo EIF4A3 bound to the WxHD motif indicates a clash with EIF4A3 residue Y202, suggesting that ALYREF can only bind to RNA-bound EJC (see Supplementary Video 2). Middle: the same view, showing the ALYREF WxHD motif bound to RNA-bound EJC (this study). Right: the same view, showing the CASC3 WxHD motif bound to RNA-bound EJC^23^, revealing conserved binding modes of ALYREF and CASC3. **d.** The ALYREF WxHD and RRM domains binds the same interfaces between EIF4A3 and MAGOH as the CASC3_SELOR_ domain. Top: Overview image of the ALYREF–EJC–RNA structure (left) and comparison of the binding modes of ALYREF and CASC3 (middle and right, respectively). Bottom: Sequence alignment of ALYREF (top) and CASC3 (bottom), showing the conserved WxHD motif and an additional short conserved motif (QEL[F/I]Ax[F/Y]G), which is however not contacting the EJC in the ALYREF–EJC–RNA structure. Conserved (dark blue) and partially conserved (light blue) residues are indicated with boxes. Residues in ALYREF and CASC3 contacting the EJC are indicated. **e.** Superposition of the ALYREF–EJC–RNA complex (this study) onto the human P-complex spliceosome cryo-EM structure (PDB ID 6QDV)^33^, via their EJC EIF4A3 subunits. This model reveals that higher order ALYREF–EJC complexes such as the ALYREF–EJC dimer are not possible when the EJC is still bound by the spliceosomes, as the P-complex subunit SNU114 clashes with the RRM in an ALYREF–EJC dimer. In addition, SNU114 likely disfavors binding of a single molecule of ALYREF to the EJC, as there is a steric clash with the N-terminal ordered ALYREF residue (Asp 85) in the ALYREF–EJC structure.

To gain further insights into how ALYREF may discriminate spliced from unspliced mRNPs^32^, we modelled our ALYREF_55-182_–EJC–RNA structure onto the mRNA-bound EJC in the human post-catalytic (P-complex) spliceosome structure^33^ or RNA-unbound EIF4A3^24^ (Extended Data Fig. 3e, Supplementary Video 2). This structural analysis revealed that only the mRNA-bound EJC can form multivalent ALYREF–EJC complexes, suggesting how pre-mRNA splicing may precede mRNP packaging.

ALYREF was also reported to associate with the mRNP-bound CBC^6^ and viral proteins, such as ORF57^34^ (Extended Data Fig. 3b). Further, other mRNA export-adaptors, such as CHTOP, LUZP4, UIF, and POLDIP3, which share no structural features with ALYREF except for the UBMs^4,29,35^, might bind mRNP features differently from ALYREF. The simultaneous binding of varied mRNA export-adaptors and ALYREF to mRNPs^4,29,36^, could thus enable the broad but specific recognition of mRNPs.

### Cryo-EM of endogenous TREX–mRNPs

**Extended Data Figure 4:**
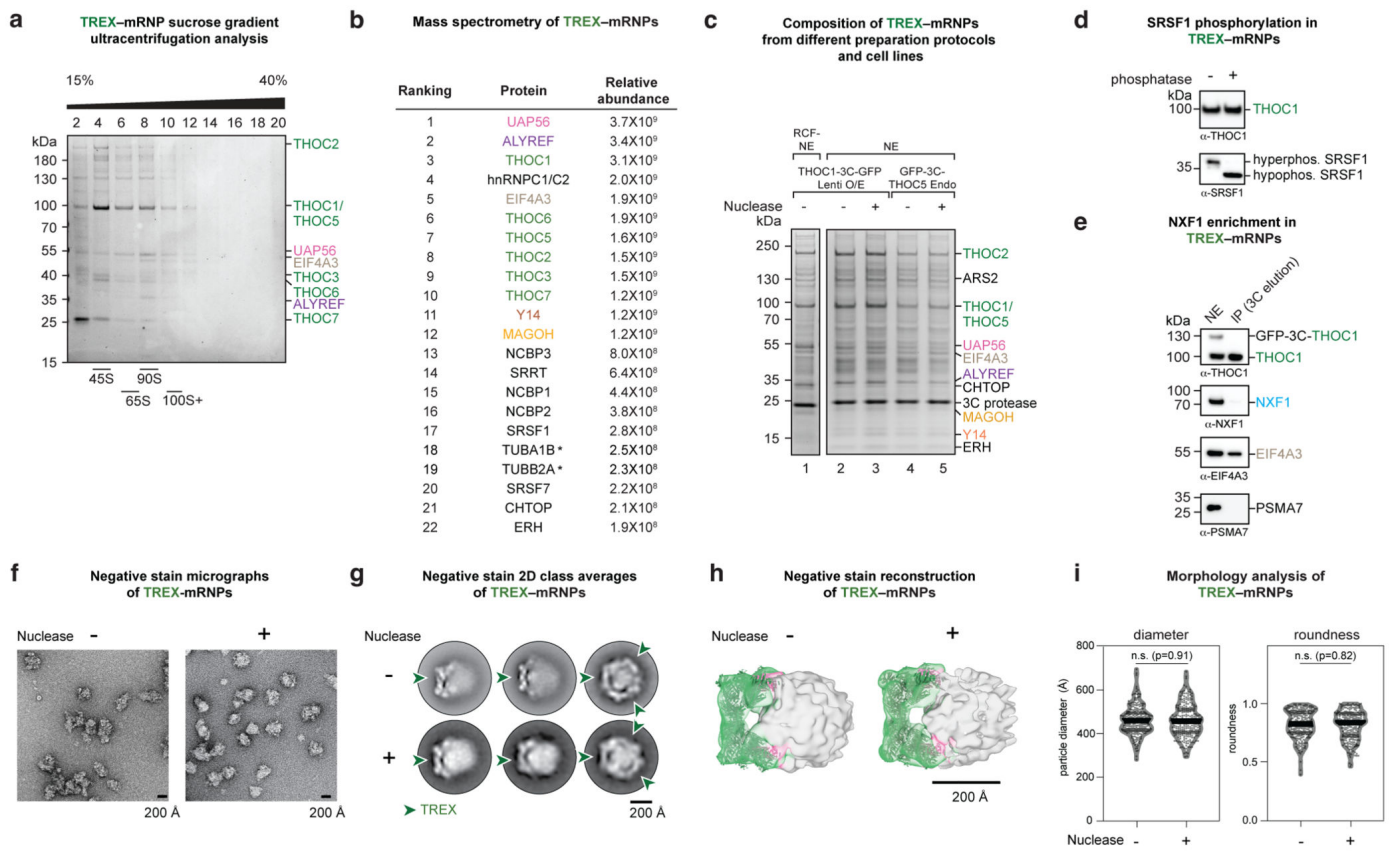
Endogenous TREX–mRNP complex purification strategies, biochemical characterization, and negative stain EM. **a.** Endogenous TREX–mRNP complexes were obtained via affinity purification of ectopically overexpressed THOC1-GFP in K562 cell nuclear extract (NE), which underwent a mild nuclease treatment. Purified TREX–mRNPs sediment ~90-100 S in a sucrose density gradient. Individual fractions were analyzed by SDS-PAGE and S-values were estimated using *CowSuite*. This experiment was done more than ten times. **b.** Mass spectrometry analysis of endogenous TREX–mRNP complexes shows the 11 members of TREX and EJC within the top 12 hits. The relative abundance of each protein was estimated by summing up the peak areas of the top three peptides. Asterisks indicate tubulin proteins, which are abundant cellular proteins that are common purification contaminants. See Supplementary Table 1 for a complete list of identified proteins. **c.** TREX–mRNP purification yields the same protein composition using different strategies: (i) two different cell lines, ectopic THOC1-GFP overexpression (Lenti O/E) versus endogenous GFP-THOC5 CRISPR/Cas9-tagging (Endo), (ii) nuclear extract preparation methods, rapid cell fractionation (RCF) versus the standard nuclear extract preparation protocol (see Methods for details) or (iii) without and with mild nuclease digestion with benzonase. SDS-PAGE gels after affinity purification using GFP-trap resin and elution with 3C protease are shown. The experiment comparing RCF versus standard nuclear extract preparation protocols was done once. The comparison between THOC1-3C-GFP Lenti O/E and GFP-3C-THOC5 Endo nuclear extracts was carried out twice. The comparison between benzonase and non-benzonase treatments was done eight times. For gel source data, see Supplementary Figure 5. **d.** SRSF1 is phosphorylated in endogenous TREX–mRNP complexes. Western blot analysis of SRSF1 in purified TREX–mRNPs before (lane 1) and after (lane 2) treatment with lambda phosphatase. Phosphorylated SRSF1 migrates slower during SDS-PAGE-PAGE and is less efficiently recognized by the anti-SRSF1 antibody. This experiment was done four times. For gel source data, see Supplementary Figure 6. **e.** NXF1 is absent from purified TREX-mRNPs. Western blot showing protein levels of THOC1, NXF1, EIF4A3 and the proteasome subunit PSMA7 control in input (standard nuclear extract) and affinity purified TREX–mRNPs. While THOC1 and EIF4A3 are enriched in TREX–mRNPs, NXF1 and the proteasome are not. NXF1–NXT1 may be absent from TREX–mRNPs either due to a low affinity interaction with TREX–mRNPs or because it associates after an additional mRNP remodelling step. The experiment was done twice. For gel source data, see Supplementary Figure 7. **f.** Mild nuclease treatment is required to obtain well-separated TREX–mRNP particles for electron microscopy. The nuclease activity of benzonase was reduced by omitting Magnesium from the buffer. Negative stain EM micrographs of TREX–mRNPs purified from nuclear extract either without (left) or with (right) mild nuclease treatment show that non-treated TREX–mRNP particles more frequently clump together. This experiment was done once. Scale bar, 200 Å. **g.** Purified TREX–mRNPs without (top) or with (bottom) mild nuclease treatment show identical negative stain EM 2D class averages. TREX complexes are indicated on the 2D classes using green arrow heads, showing that in both conditions single and multiple TREX complexes bound to a globular mRNP density. Scale bar, 200 Å. **h.** Purified TREX–mRNPs without (left) or with (right) mild nuclease treatment show identical negative stain EM 3D reconstructions. Scale bar, 200 Å. **i.** Nuclease treatment does not affect TREX–mRNP particle diameter or shape when visualized with negative stain EM. Left: Violin plot of TREX–mRNP particle diameters measured on negative stain electron micrographs. Horizontal bars indicate 25^th^ (grey), 50^th^ (black) and 75^th^ (grey) percentiles. Nuclease-treated (n=259) or untreated (n=245) particles are not significantly different (Welch’s t-test, p=0.91). Right: Particle roundness, calculated by dividing the length of the shortest axis of each particle by the length of the longest axis, is also not significantly different (Welch’s t-test, p=0.82).

**Figure 2:**
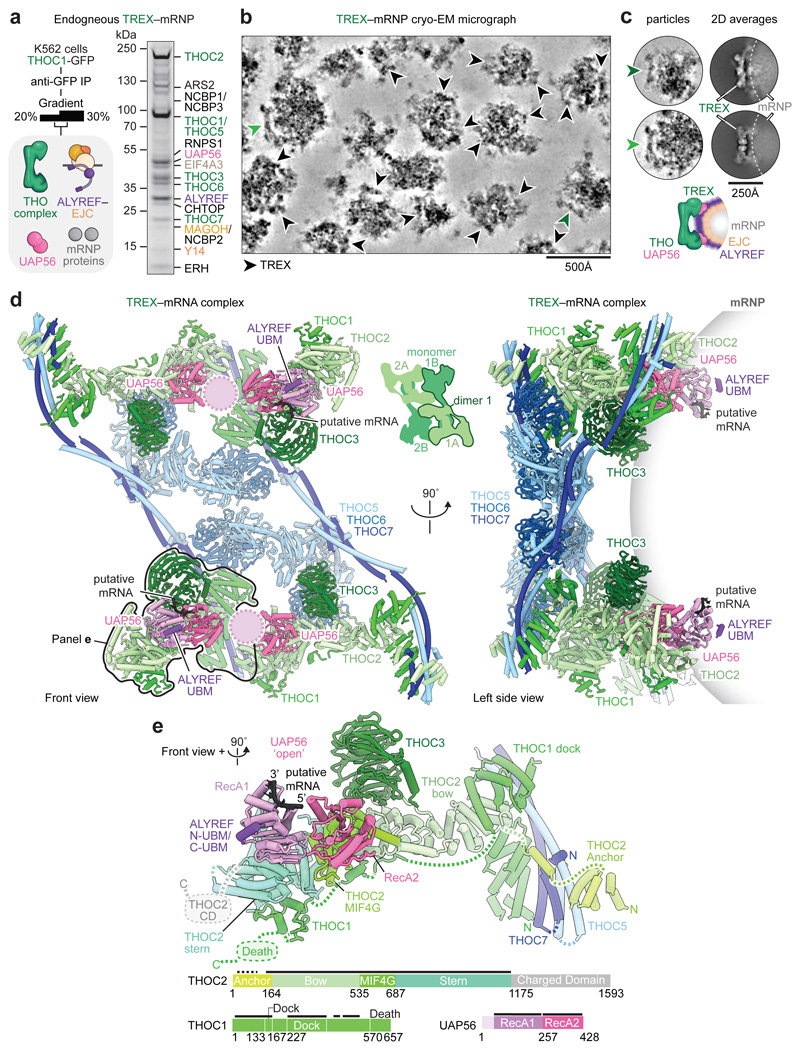
Endogenous TREX–mRNP complex structure. **a.** Purification scheme (left) and SDS-PAGE analysis (right) of endogenous TREX-mRNP complexes from human K562 cells (see Methods, Extended Data Fig. 4). For gel source data, see Supplementary Figure 2. **b.** Denoised cryo-EM micrograph of TREX–mRNPs (see Methods). TREX complexes are indicated with arrows heads. Scale bar, 500 Å. **c.** Single cryo-EM particles of TREX complexes on TREX–mRNPs are shown (left) next to corresponding 2D averages (right). A curved dashed line (white) separates TREX from mRNP densities in the 2D averages. A schematic of the 2D averages is shown underneath with protein colors as in panel **a**. **d.** TREX–mRNA structure shown from front and left side views. The UAP56 RecA1 (light pink) and RecA2 (pink) domains are observed in TREX monomer B, while monomer A is more mobile, precluding structural modelling of the UAP56 RecA1 lobe (see Extended Data Fig. 5d, g, h). Since ALYREF N- or C-terminal UAP56-binding motifs (UBMs) cannot be distinguished at low resolution, we label these peptides as ‘UBM’, and modelled them based on the C-UBM in the homologous yeast Sub2–Yra1–RNA crystal structure^41^ and AlphaFold2 multimer. In the inset (top center) TREX–mRNA complex dimers are labelled 1 and 2, and the constituent monomers A and B. THOC1 (green), -2 (light green), -3 (dark green), -5 (light blue), -6 (blue), -7 (light blue), UAP56 RecA1, -2 (light pink, pink), ALYREF (purple), putative mRNA (black). **e.** Details of the TREX–mRNA structure. THOC2 is colored in shades of green, other subunits and mRNA colored as in **d**. THOC6, and THOC5 and -7 C-terminal regions were omitted for clarity (see also Extended Data Fig. 5d, g, h). Domain organization (bottom) of THOC1, THOC2, and UAP56. Solid and dashed black lines indicate atomic and backbone regions, respectively. CD, charged domain; Death, death domain.

Our recombinant TREX–EJC–RNA data suggested models for mRNP recognition and TREX assembly, but how endogenous mRNPs organize in three dimensions and how they engage complete TREX complexes remained unclear. To investigate these questions, we purified endogenous TREX–mRNP complexes from human cells and analyzed these using cryo-EM and protein crosslinking. For purification, we overexpressed the GFP-tagged TREX subunit THOC1 in human K562 cells^28^ and isolated TREX–mRNPs from nuclear extract. TREX–mRNPs sedimented at ~90S (Extended Data Fig. 4a) and contained all TREX complex subunits, the EJC and additional mRNP components such as the CBC, CHTOP, ERH, and SRSF1 (Fig. 2a, Extended Data Fig. 4b, Supplementary Table 1), consistent with previous purifications of human TREX and spliced mRNPs^6,37^. TREX–mRNPs contained phosphorylated SRSF1 (Extended Data Fig. 4d), a marker of nuclear mRNPs^38^, but lacked the mRNA export factor, NXF1–NXT1 (Extended Data Fig. 4e). We obtained the same TREX–mRNP protein composition from two additional purification strategies, using either a different nuclear extract preparation procedure or a CRISPR-Cas9 GFP knock-in on the THO subunit THOC5 in K562 cells, supporting the robustness of our approach (Extended Data Fig. 4c, Methods). mRNA 3’-end sequencing of purified TREX–mRNPs revealed a large diversity of human mRNAs, in agreement with ALYREF iCLIP data^8,13^ (Supplementary Table 2). Taken together, we were able to isolate endogenous mRNPs bound to the complete TREX complex, prior to loading of the mRNA export factor.

**Extended Data Figure 5:**
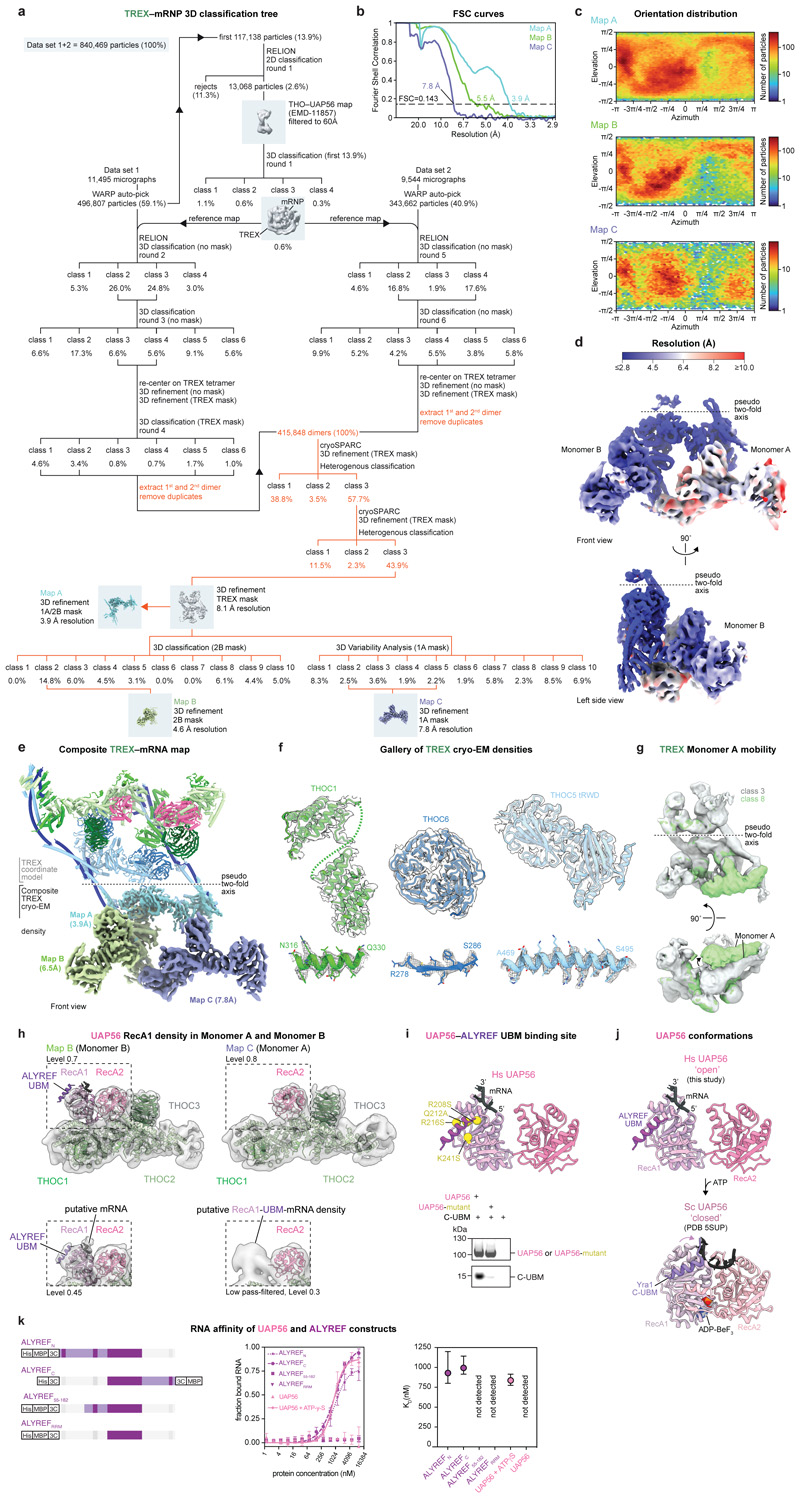
Endogenous TREX–mRNP complex cryo-EM image processing, reconstructions, and biochemistry of UAP56–ALYREF. **a.** Three-dimensional image classification tree of endogenous TREX–mRNP complex cryo-EM data^28,65^. The complete data set contained 840,469 TREX-mRNP particles, which were classified in multiple rounds of 3D classification (with regularization parameter T=4 for all RELION classifications) and focused refinement in RELION^66,67^. The best particles were used to extract symmetry related dimers, separately, yielding 415,848 dimer particles, which were further classified and refined in cryoSPARC^64^. This yielded maps A (cyan), B (light green), and C (slate blue) (see Methods for details). The percentage of TREX–mRNP particles (black) or TREX dimer units (orange) contributing to each class are provided. The type of mask and overall resolution is indicated for each 3D refinement. **b.** Gold-standard Fourier shell correlation (FSC = 0.143) of the TREX–mRNA cryo-EM maps A, B, and C. **c.** Orientation distribution plots for all particles contributing to the TREX–mRNA cryo-EM maps A, B, and C, visualized in cryoSPARC^64^. **d.** The composite TREX–mRNA cryo-EM density is shown from front and left side views (maps A, B, and C), and colored by local resolution as determined by cryoSPARC^64^. **e.** The composite TREX–mRNA cryo-EM density (maps A, B, and C) is shown opposite of the refined TREX–mRNA coordinate model, which is shown as ribbons and colored as in Fig. 2d. **f.** Gallery of TREX–mRNA complex subunits THOC1, THOC5 (tRWD domain), and THOC6 are shown superimposed on their respective cryo-EM densities. Below each protein a representative segment of the protein is superimposed on the respective cryo-EM density. **g.** The TREX monomer A is mobile in the TREX–mRNA complex data. Two densities obtained from 3D variability analysis (class 3 in grey and class 8 in green) are overlayed, revealing that monomer A can shift globally by ~25 Å. This mobility can explain why monomer A, and the associated UAP56 molecule, have a low local resolution. **h.** The TREX–mRNA map reveals density for the UAP56 RecA1 lobe, the ALYREF UBM, and putatively assigned mRNA, which were fitted as a single rigid body of a yeast Yra1–Sub2–RNA homology model (5SUP). The ALYREF UBM, which could be either N- or C-terminal, is visible at lower density threshold, and was modelled as the C-UBM based on its position in the yeast Yra1 (C-UBM)–Sub2–RNA crystal structure and an AlphaFold2 mulitmer model^62^ of the ALYREF C-UBM bound to human UAP56. **i.** Mutation of human UAP56 residues at the ALYREF-UBM to UAP56 interface, supports the ALYREF-UBM density assignment. Top: Interface mutations are mapped onto the UAP56 coordinate model and labelled. Bottom: *In vitro*, a fluorescently labeled ALYREF C-UBM peptide binds to wildtype UAP56 but not mutated UAP56. This experiment was done once. For gel source data, see Supplementary Figure 8. **j.** Comparison of human ALYREF-UBM–UAP56–RNA (this study) and yeast Yra1-UBM–UAP56–RNA–ATP-analog (5SUP)^41^ structures. **k.** An RNA filter-binding assay suggests that the ALYREF RNA binding domains 1 and 2 (RBD1 and RBD2) might assist RNA delivery to UAP56, but not the isolated ALYREF_55-182_ construct that forms EJC contacts (see Fig. 1, Extended Data Fig. 1). Left: Boundaries of protein constructs used for RNA affinity measurements using filter binding assays. Middle: Binding curves of the tested constructs. The plot shows mean values from n=6 measurements, error bars indicate the standard deviation of each measurement, and solid or dotted lines show the fit of a “Specific binding with Hill-slope”-function to the data, with the Bmax constrained to 1 as implemented in GraphPad Prism (see Methods). Right: Measured dissociation constants (KD) of the tested constructs as determined by the fits in the middle panel; spheres indicate the KD determined form the fit and error bars indicate the 95% confidence interval determined from the fit. UAP56-RNA binding is not detectable with isolated UAP56 in absence of ATPγS, but does bind RNA with K_D_ of ~900 nM (95% confidence interval: 810-1,014 nM) in presence of 1 mM ATPγS. The ALYREF-RNA binding activity is contained in its RBD1 and RBD2 domains, but not in the WQHD or RRM domains. These experiments were done twice, with three technical replicates each.

For cryo-EM imaging we subjected TREX–mRNPs to a mild nuclease treatment, which was necessary to obtain well-separated TREX–mRNP particles on cryo-EM grids without altering their protein composition, protein stoichiometry, three-dimensional architecture, or average particle diameter (Fig. 2b, c, Extended Data Fig. 4c, f-i). While yeast^39^ and insect Balbiani ring^40^ mRNPs were previously visualized at low resolution and in non-native conditions, the cryo-EM micrographs presented here show endogenous human mRNPs in native conditions (Fig. 2b). TREX–mRNPs vary in their overall dimensions (median diameter of ~450 Å, ranging from 300-700 Å), likely due to the diverse mRNAs associated with TREX at steady state (Fig. 2b, Extended Data Fig. 4f, i, Supplementary Table 2). Reference-free, two-dimensional classification revealed TREX complex densities on the mRNP surface, while mRNP densities were diffuse (Fig. 2c). To understand how TREX engages mRNPs, we processed the cryo-EM data to obtain a composite TREX density at nominal resolutions between 3.9-7.8 Å (maps A, B, C) (Extended Data Fig. 5a-f, Methods). [Fig F3]

### TREX–mRNP structure and contacts

**Extended Data Figure 6:**
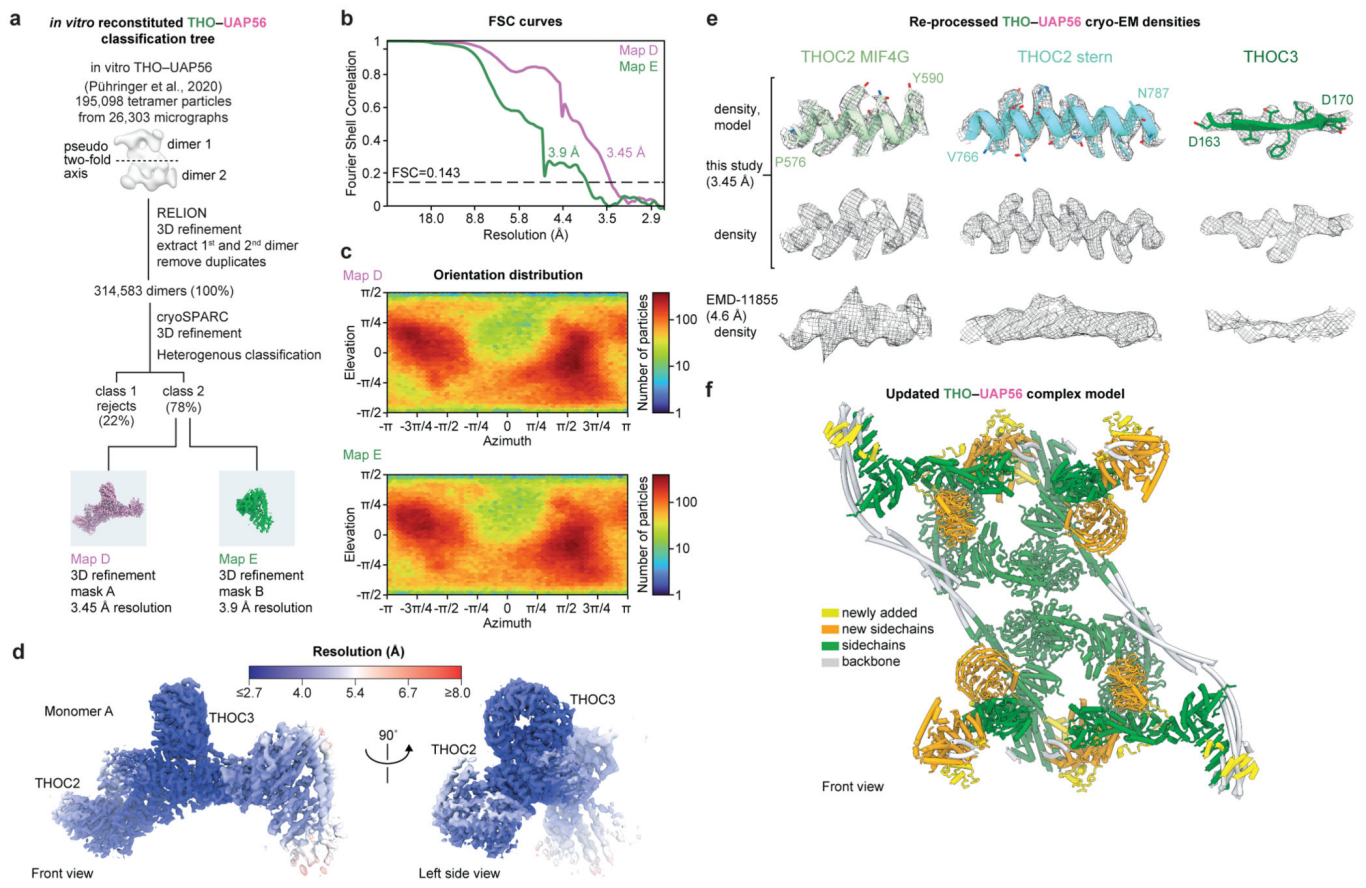
Recombinant THO–UAP56 complex cryo-EM image processing and reconstructions. **a.** Three-dimensional image classification tree of the *in vitro* reconstituted THO–UAP56 cryo-EM data set^28^. The symmetry-expanded data set contained 314,583 high-quality particles. Classification and focused refinements in cryoSPARC ^64^ yielded maps D (pink) and E (green) (see Methods for details). The percentage of THO–UAP56 dimer units contributing to each class is provided. The type of mask and overall resolution is indicated for each 3D refinement. **b.** Gold-standard Fourier shell correlation (FSC = 0.143) of the cryo-EM maps D and E. **c.** Orientation distribution plots for all particles contributing to cryo-EM maps D and E, visualized in cryoSPARC^64^. **d.** THO–UAP56 complex monomer A composite cryo-EM densities from front and left side views (maps D and E), colored by local resolution as determined by RELION 3.1^66^,^67^. **e.** Representative regions of the newly determined THO–UAP56 cryo-EM densities (top) in comparison to previous data (bottom)^28^. The new densities are superimposed on the updated and refined THO–UAP56 coordinate model. Segments of THOC2 residues 316-330, residues 576-590, and THOC3 residues 163-170 are shown. **f.** A new model of the human THO–UAP56 complex. Newly modelled regions are shown in yellow, and contain segments of THOC1, THOC2, and THOC3. Regions with newly modelled sidechains are colored orange and are built on the previously available backbone models of THOC2 and THOC3. This updated model reveals new contacts among THOC1, -2, and, -3 subunits. The newly built THOC1 C-terminus meanders along the length of the THOC2 subunit ‘bow’, ‘MIF4G’, and ‘stern’ domains (Fig. 2e). The THOC1 C-terminal residues (458-528) were initially modelled using AlphaFold (Methods)^62,63^. The THOC2 ‘anchor’ forms a 5-helix bundle that packs against THOC5 helix α2 and THOC7 helices α2 and α3, and the THOC3 β-propeller blades 3 and 4 make a stabilizing contact with THOC2 ‘bow’ loop α17-α18 (Fig. 2e). Unchanged regions are colored grey and green and contain modelled backbones or sidechain, respectively.

The human TREX core THO–UAP56 complex forms a tetramer and comprises two asymmetric dimers, 1 and 2, that each contain two seven-subunit monomers A and B^28^ (Fig. 2d). The recombinant THO–UAP56 structure^28^ showed an excellent fit to the native TREX cryo-EM maps and revealed additional densities near THO complex subunits THOC2, THOC3, and UAP56. To determine the identity of these densities, we first re-processed our previous recombinant THO–UAP56 cryo-EM dataset^28^ to prepare a highresolution model of THO–UAP56. We improved monomer A densities from 4.6 to 3.5 Å resolution (maps D, E) (Extended Data Fig. 6a, b), from which we derived an updated and near-complete model of the entire 28-subunit THO–UAP56 complex. We newly built structures of the THOC1 C-terminus and THOC3 subunit, and updated THOC2 ‘anchor’, ‘bow’, MIF4G, and ‘stern’ domains (Fig. 2e, Extended Data Fig. 6f). UAP56 contains two lobes, RecA1 and RecA2. The new THO–UAP56 model explained the majority of the endogenous TREX density and contained the UAP56 RecA2 lobe in monomer A and B. The remaining density was in monomer B and belonged to UAP56 RecA1, an ALYREF UBM (N- or C-terminal), and putatively assigned mRNA (Extended Data Fig. 5h), which we fitted using a homology model of the yeast UAP56–RNA–ALYREF crystal structure^41^. While we also observed additional density in the equivalent location in monomer A, we did not model the UAP56 RecA1 lobe, ALYREF, and putative mRNA owing to the low local resolution (Extended Data Fig. 5d, h). The final native TREX–mRNA model contains 30 proteins (Fig. 2d, Supplementary Video 4, Extended Data Table 1). [Fig F4]

In the structure, the 2 MDa TREX complex contacts endogenous mRNPs exclusively through its UAP56 and ALYREF subunits, which also bind to each other (Fig. 2d). UAP56 (monomer B) assumes an open conformation, consistent with the absence of ATP in the sample and with yeast THO–Sub2 (human UAP56) cryo-EM structures^5,31,41^ (Extended Data Fig. 5j). The contacts of UAP56 RecA1 and RecA2 lobes with the THOC2 ‘stern’ and ‘MIF4G’ domains, respectively, can explain how THO stimulates UAP56 ATPase activity, by promoting UAP56 opening^28,42^. ALYREF binds UAP56 exclusively through its UBMs, consistent with *in vitro* data from yeast and human systems (Extended Data Fig. 1m). In support of the ALYREF UBM assignment, mutation of the human UAP56–ALYREF UBM interface impairs their interaction *in vitro* (Extended Data Fig. 5i) and a human UAP56–ALYREF C-UBM AlphaFold model predicts the same binding site as observed in the yeast UAP56–ALYREF C-UBM crystal structure (Fig. 5j, Methods). Both UBMs are required for normal ALYREF function^43,44^ and connect to RNA-binding domains RBD1 or RBD2, respectively. The RBDs are disordered in our structure (Fig. 2d, Extended Data Fig. 3a), but may help deliver the mRNA to UAP56. Supporting this model, the isolated UAP56 does not bind RNA in absence of ATPγS, whereas ALYREF_N_ binds RNA with moderate affinity (~0.6 μM) (Extended Data Fig. 5k). The isolated yeast ALYREF (Yra1) N- and C-UBMs bind yeast UAP56 (Sub2) with low affinity (~2.9 μM each)^31^, which can explain why isolated human ALYREF_N_, containing only one UBM, does not form a stable complex with THO–UAP56 *in vitro* (Extended Data Fig. 1k). Despite these low affinities, endogenous TREX complexes stably bind mRNPs (Fig. 2a-c). Because human mRNAs contain on average eight splice junctions^19^, they could bind up to eight EJCs^1^ and thus eight ALYREF molecules (16 UBMs), assuming equimolar binding of ALYREF to the EJC. Considering average TREX–mRNP diameters of ~450 Å (Extended Data Fig. 4i), this would result in a high UBM concentration (~560 μM, see Methods) in mRNPs and thereby could promote efficient THO–UAP56 recruitment.

**Figure 3 F3:**
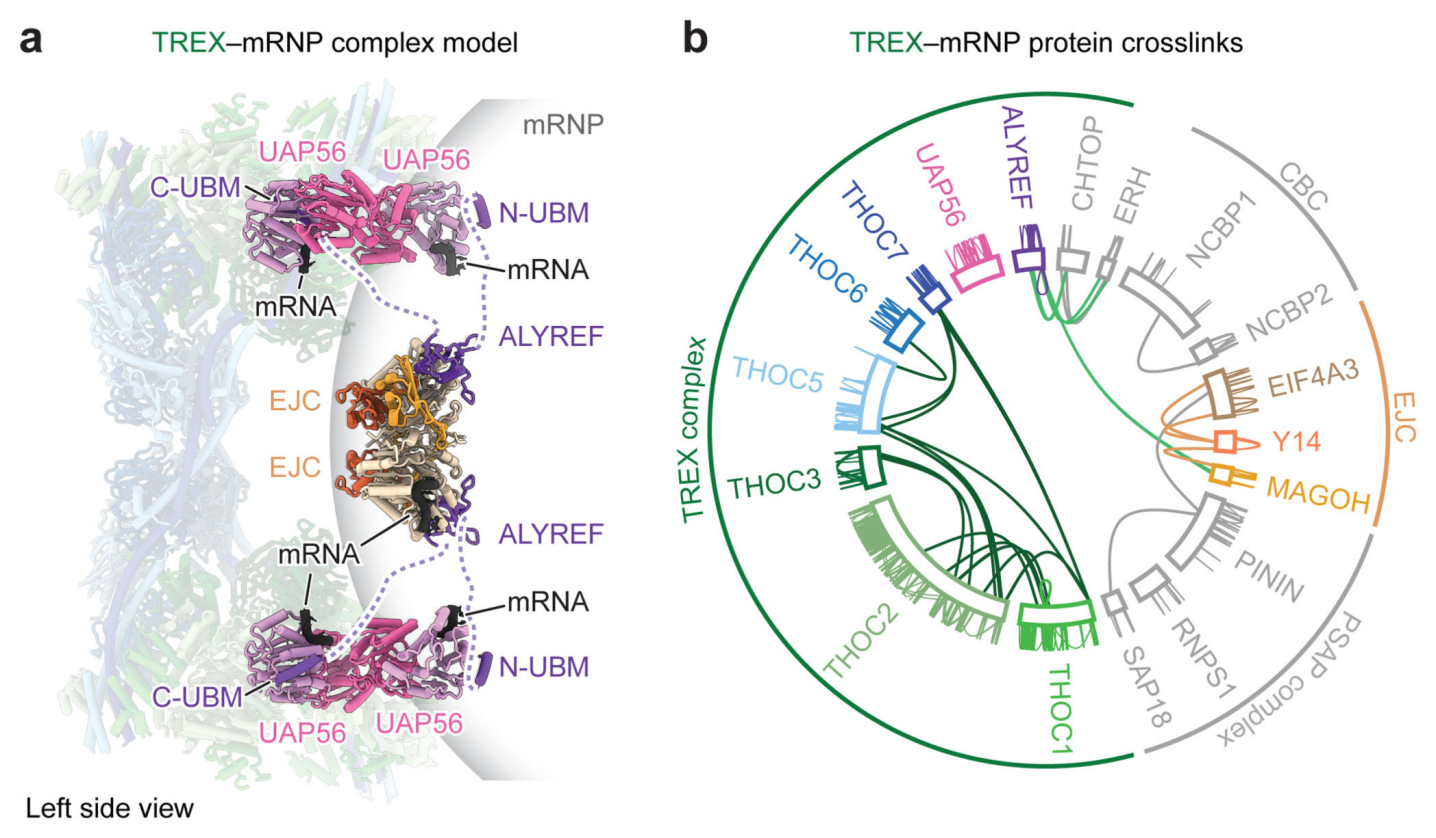
TREX–mRNP model and protein crosslinking. **a.** The TREX–mRNP complex model in a left side view shows how ALYREF could simultaneously (i) recognize the mature mRNP through its WxHD and RRM domains, (ii) bind adjacent UAP56 helicases through its N- and C-UBMs, and (iii) guide mRNA to UAP56 through its RBD1 and -2 domains. Note that N- and C-UBM assignments show one of many possible arrangements and in native complexes not all ALYREF UBMs would need to be engaged with THO–UAP56. The shown model illustrates the concept of multivalent ALYREF UBM to UAP56 interactions (see Supplementary Video 5). To obtain this model, we superimposed the UAP56–ALYREF-mRNA model from TREX monomer B on monomer A. The THO complex is shown as a transparent cartoon. An ALYREF–EJC–RNA dimer was placed into the mRNP density. **b.** TREX–mRNP protein-protein crosslinks agree with the model. 3,125 crosslinked residues were detected using the UV-activatable crosslinker sulfo-SDA (2% protein-protein interaction-level false discovery rate). Inter- and intra-protein crosslinks are shown for selected TREX–mRNP proteins (see Extended Data Fig. 7, Supplementary Table 3). Crosslinks within TREX are shown in dark green lines, crosslinks from ALYREF to mRNP proteins in light green, within the EJC in orange, and within mRNP proteins in grey. Protein colors as in panel **a**.

Additional ALYREF–CBC interactions^6^ and other export-adaptors could further increase the UBM concentration at mRNPs. This could also explain how mRNPs, which are generated from the five percent of human protein-coding genes lacking introns^19^ and thus lacking EJCs, may accumulate UBMs. The flexible ALYREF domain organization may further enable the TREX–mRNP interaction to engage mRNPs of various sizes. Together, these findings mirror our observations with *in vitro* reconstituted TREX–EJC– RNA complexes (Extended Data Fig. 1k-m), and suggest that TREX assembles through many low-affinity interactions of THO–UAP56 with multiple molecules of mRNP-bound ALYREF or other export-adapters (Fig. 3a, b, Supplementary Video 5).

### A multivalent TREX–mRNP model

**Extended Data Figure 7:**
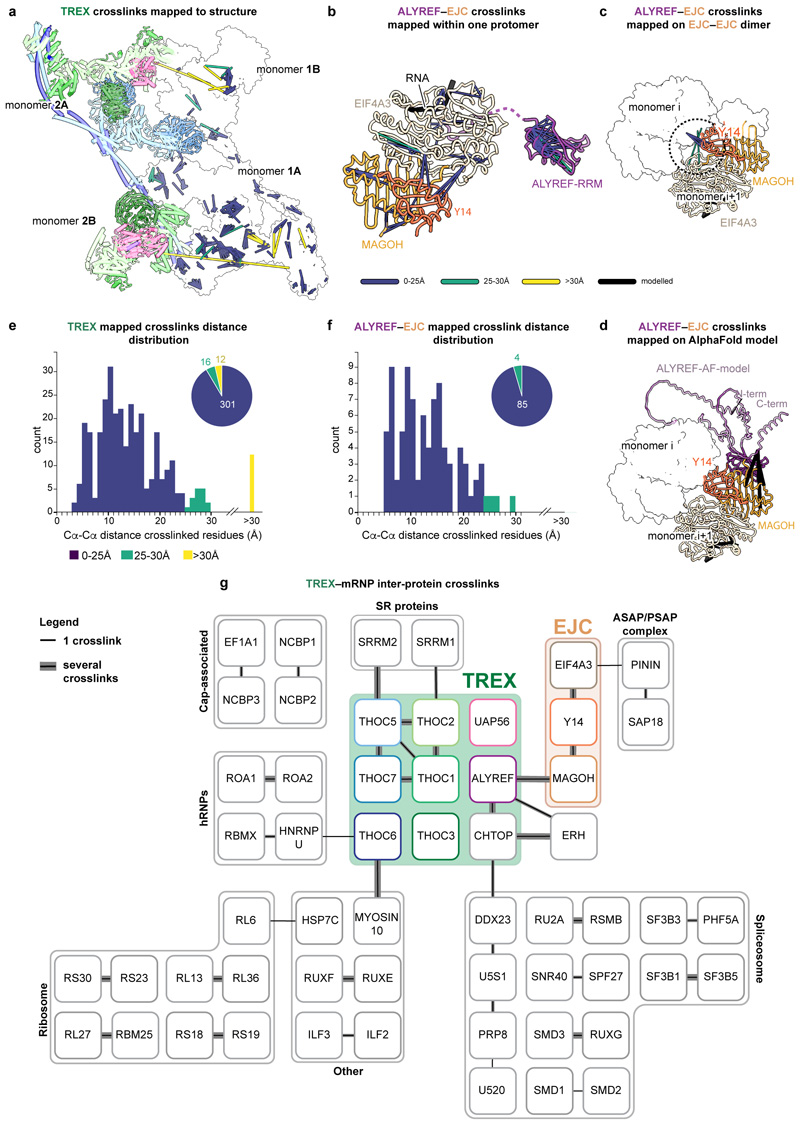
Crosslinking MS of endogenous TREX–mRNPs. **a.** Crosslinks mapped onto TREX monomers 1A and 1B. Monomer 1A and 1B are shown as transparent surfaces and crosslinks are colored according to the Cα-Cα distance of crosslinked residues. Symmetry related monomers 2A and 2B are shown in ribbon representation and colored as in Fig. 2d. Crosslinks that span more than 30 Å may be explained through proximity between TREX complexes on mRNPs, as observed in our cryo-ET data. The data was generated from two purification and crosslinking experiments, which were merged for data analysis (see methods). **b.** Crosslinks mapped onto the ALYREF–EJC–RNA protomer structure. **c.** Crosslinks mapped onto the ALYREF–EJC–RNA dimer structure are similarly compatible both with inter EJC-EJC (dimer) as well as with intra–EJC crosslink distances (protomer, panel **b**). Crosslinks spanning less than 30 Å are shown. **d.** The ALYREF–MAGOH crosslinks mapped onto a model generated by superposing the ALYREF AlphaFold model onto the ALYREF-RRM. ALYREF residues in the AlphaFold model that are absent from the ALYREF–EJC–RNA structure are shown as transparent ribbons. **e.** Histograms and pie charts of Cα-Cα distances of crosslinked residues in the TREX (**e**) structure. **f.** As panel **e**, but for the ALYREF–EJC–RNA structure. **g.** Protein-protein interaction network based on crosslinks of TREX–mRNPs after a one-step purification without nuclease digestion. Note that ribosomal proteins are common contaminants. The thickness of the grey lines connecting proteins scales with the number of unique crosslinked residue pairs.

To further explore this structural model of TREX–mRNP interactions (Fig. 3a), we carried out crosslinking coupled to mass-spectrometry (crosslinking MS). We applied a UV-activated crosslinking agent to obtain specific crosslinks within native TREX–mRNPs, from which we obtained 3,125 crosslinked residues pairs (Fig. 3b, Extended Data Fig. 7, Supplementary Table 3). Intra- and inter-protein crosslinks within TREX and the EJC are in excellent agreement with our cryo-EM structures, demonstrating the high quality of the crosslink MS data (Extended Data Fig. 7a-f). We further detected crosslinks between ALYREF and the EJC, confirming that the ALYREF–EJC interaction reconstituted *in vitro* also occurs in native complexes (Fig. 3b). In addition, we identified crosslinks within the CBC and between the EJC EIF4A3 subunit and the PSAP complex subunit PININ (Extended Data Fig. 7g), in agreement with their reported interactions^47,48^. Crosslinks between multiple ALYREF molecules and of ALYREF with the alternative export-adapter CHTOP and the protein ERH (Fig. 3b, Extended Data Fig. 7g) suggest that the various export-adaptors could act cooperatively rather than independently. Collectively, these interactions would result in multivalent TREX–mRNP assembly.

### TREX–mRNP cryo-tomography

**Extended Data Figure 8:**
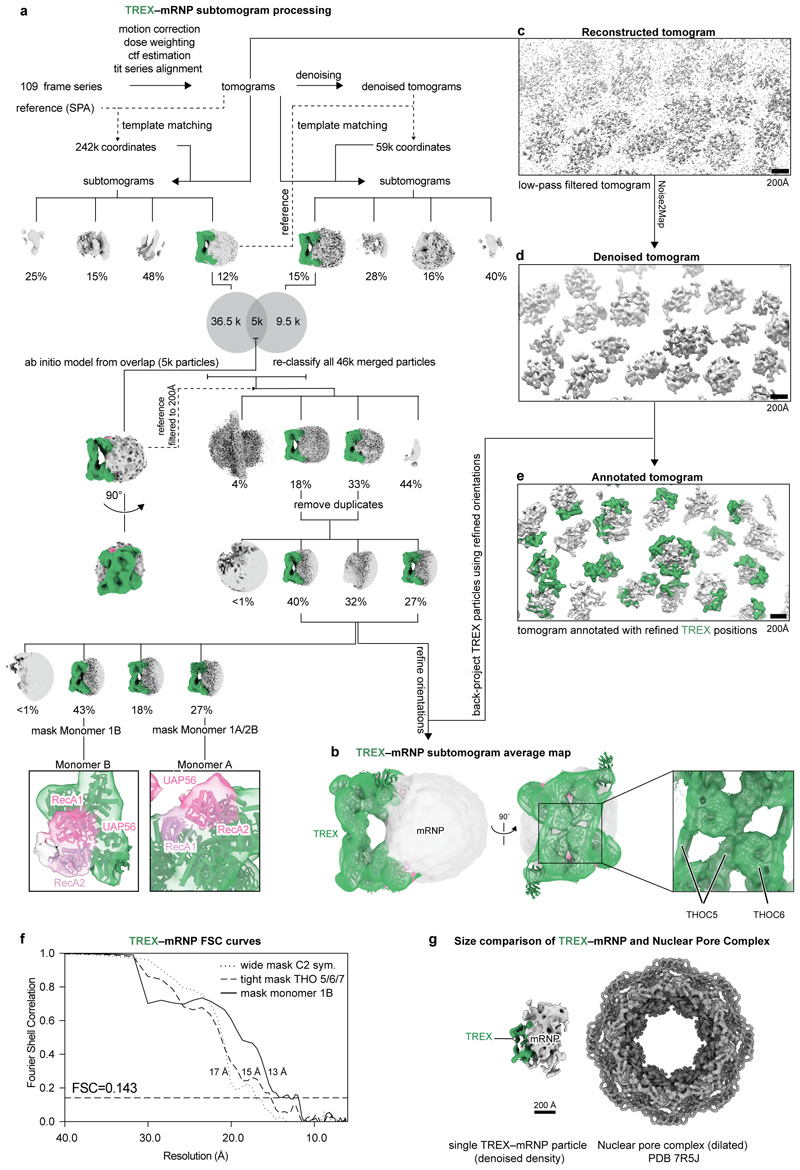
TREX–mRNP cryo-tomography analysis. **a.** Tilt-series pre-processing, tomogram reconstruction, template matching and particle classification. Tilt series movie frames were pre-processed using *Warp*^49,68^ and aligned in *imod^69^* and tomograms were reconstructed in *Warp* with a pixel size of 10 Å/px (see methods for details). Template matching and subtomogram reconstruction were performed in *Warp*. Two independent rounds of template matching and particle classification were performed; for the first round (left hand side), template matching was performed against raw tomograms using a reference volume from our single particle analysis of the endogenous TREX complex (this study). 242,237 subtomograms were extracted and classified into four classes using RELION^66,67^, and the regularization parameter was set to T=4 for all classification runs. The best class (12% of extracted subtomograms) was denoised and used to perform template matching with denoised tomograms as search targets (right branch). This yielded 59,275 subtomograms, and particle classification was performed as before. In the next step, the overlap of good particles from both branches was taken as a high-confidence set and these particles were used to generate a reference-free volume to exclude potential reference bias in the final reconstruction. The obtained volume was used to further classify the combined particles from both picking strategies using three subsequent rounds of 3D classification. In the last round, a combined 10,105 sub-tomograms in classes 2, 3, and 4 contained the TREX complex and less then 1% of particles (class 1) gave rise to ‘junk’ particles, showing that classification had converged. The insets show zoom-ins of two classes that reveal unambiguous, low-resolution density for the UAP56 RecA1 lobe (monomer B or monomer A, respectively). **b.** Subtomogram average (STA) map of endogenous TREX–mRNPs with TREX density in green and mRNP density in grey. Insets show zoom-ins on the THO complex scaffold subunits (THOC5, -6, and -7), revealing an excellent fit of the TREX structure to the STA map and density features consistent with the resolution estimate (13 Å), such as the “hole” in the THOC6 WD40 density. **c.** Example of a reconstructed tomogram before denoising. **d.** The same tomogram as shown in panel **c** after denoising. **e.** The same tomogram as in panel **d**, but with TREX positions (green densities) obtained from STA overlayed. **f.** Gold-standard Fourier shell correlation (FSC = 0.143) curve for the STA reconstruction with three different masks: (1) either a wide mask encompassing the C2 symmetric entire TREX complex (dotted line, 17 Å), (2) a tight mask encompassing the “scaffold” made from THOC5, -6, and -7 (15 Å), or (3) a tight mask around monomer B (THOC 1/2/3) (13 Å). **g.** Size comparison between a representative TREX–mRNP and the dilated human nuclear pore complex (PDB 7R5J). Visually identified TREX density in the TREX–mRNP particle is colored green, and mRNP density is colored grey.

**Extended Data Figure 9:**
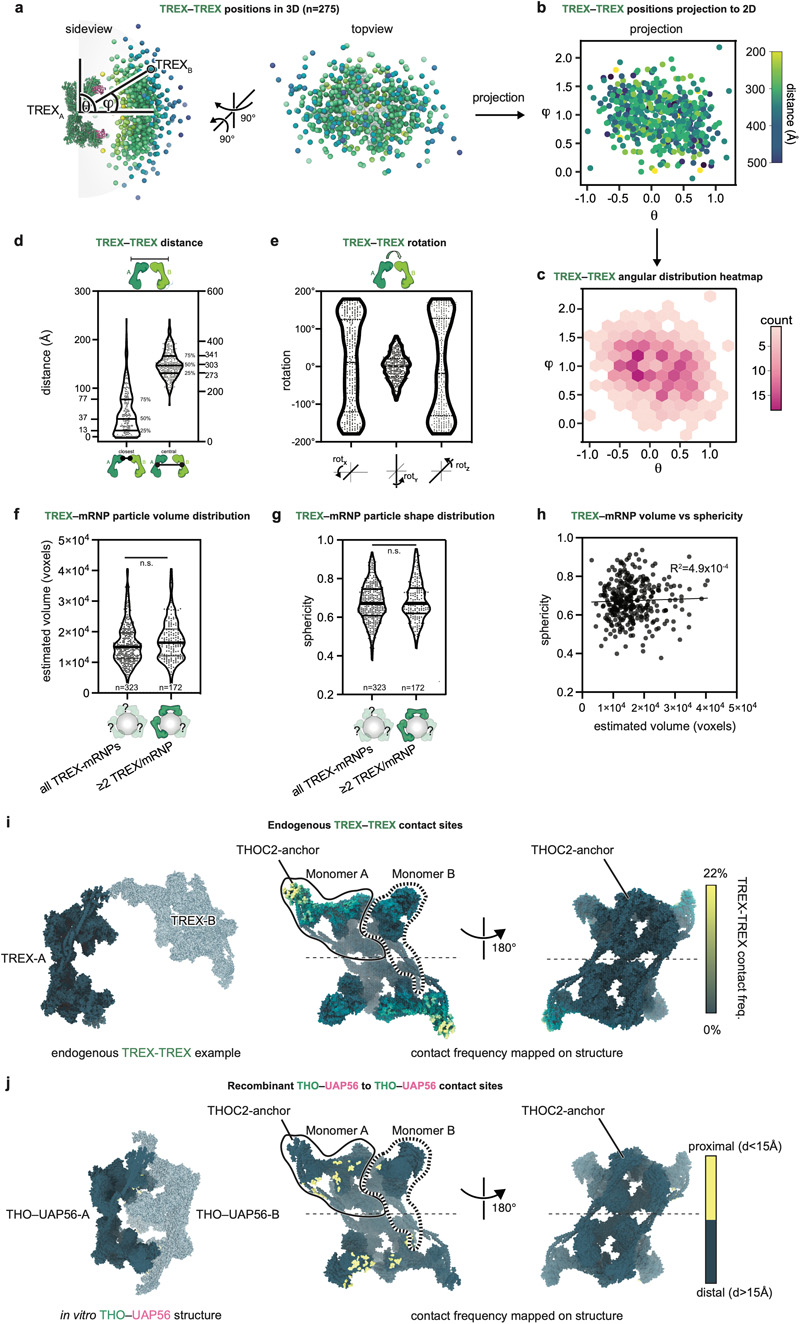
Analysis of TREX-pairs on mRNPs. **a.** Real-space representation of aligned TREX pairs (n=275) shown from two views. The reference TREX (TREX-A) is shown as a ribbon representation, and all TREX-Bs are shown as a sphere placed at the TREX-B center. Spheres are colored by TREX-A to -B distances. **b.** Projection of TREX-B coordinates onto a 2D plane, colored as in **A**. θ and φ describe the angular component of a vector connecting TREX-A with TREX-B. **c.** Heatmap of TREX–TREX positions (expressed as θ and φ). **d.** Violin plot of TREX-A–TREX-B distances, measured from center-to-center or between the two closest atoms. **e.** Violin plot of rotation angles around the X, Y and Z axis that would align TREX-A with TREX-B. **f.** Violin plot of TREX mRNP particle volumes measured for particles with more than two TREX complexes per mRNP in our stringently classified dataset or of random TREX–mRNP particles. No significant difference was found (Welch’s t-test, p=0.0874). **g.** Violin plot of TREX mRNP particle sphericity measured for particles with more than two TREX complexes per mRNP in our stringently classified dataset or of random TREX–mRNP particles. No significant difference was found (Welch’s t-test, p=0.3162) **h.** Scatter plot of TREX–mRNP volume vs sphericity (n=323). **i.** Analysis of TREX-A to -B contacts (defined as atoms of TREX-A within 10Å to TREX-B) as observed for TREX pairs on endogenous mRNPs. TREX residues are colored by their proximity frequency, with atoms never in proximity to TREX-B in bluegreen and atoms frequently in bright yellow. **j.** Analysis of THO–UAP56 contact sites (defined as atoms of THO–UAP56-A within 10 Å to THO–UAP56-B) as observed for the *in vitro* THO–UAP56 structure^28^. Atoms within 15 Å to the second copy are colored bright yellow.

**Figure 4 F4:**
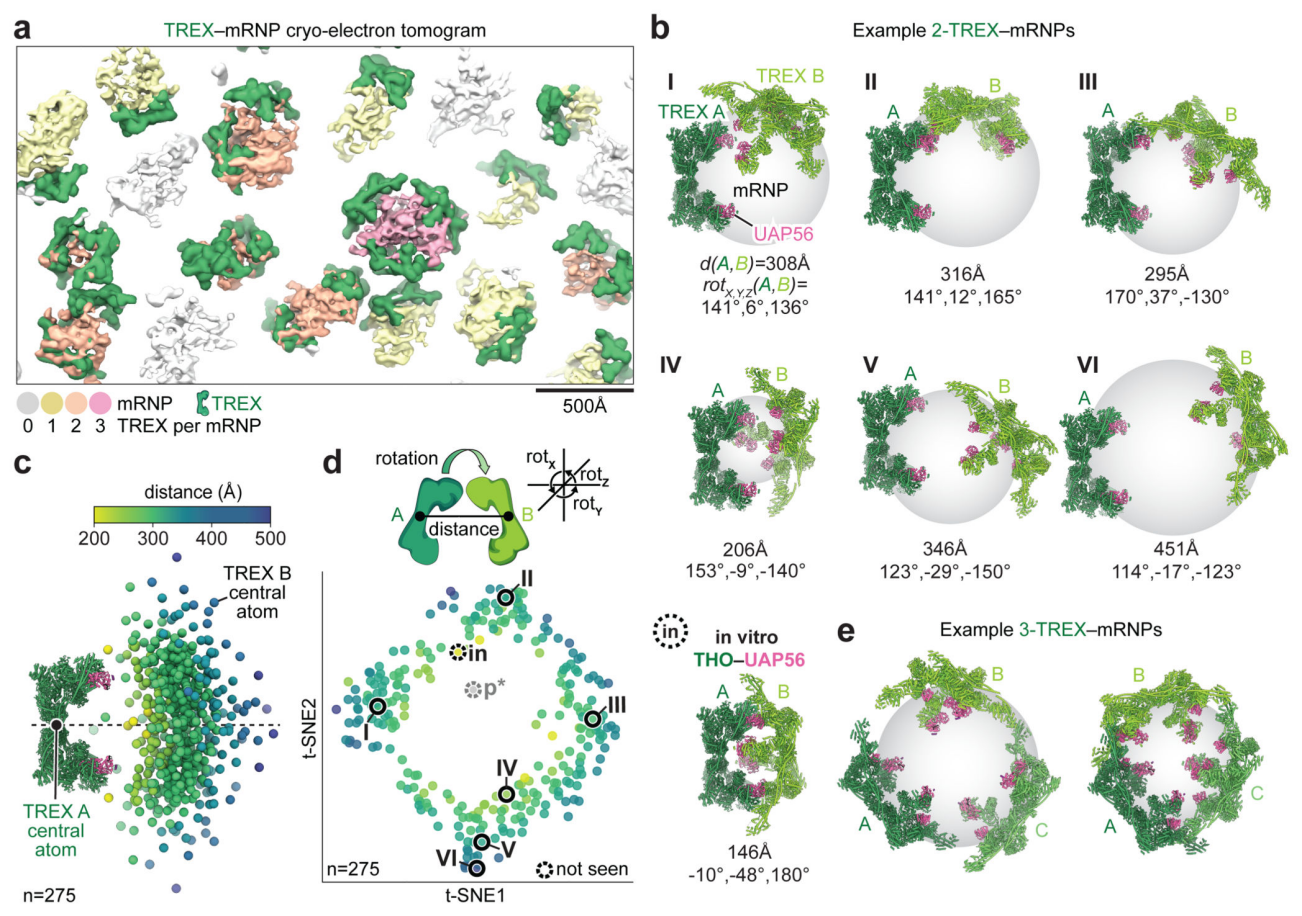
TREX–mRNP complex architectures. **a.** Isosurface representation of a denoised TREX–mRNP cryo-EM tomogram with annotated TREX complexes colored in green (see Methods and Extended Data Fig. 8). mRNP densities contain one, two, three, or no high-confidence TREX complexes and are colored in yellow, orange, pink, and grey, respectively. Scale bar, 500 Å. **b.** Gallery of TREX–mRNPs containing two TREX complexes. Examples I-VI show selected TREX–mRNPs with TREX complexes A (dark green) and B (light green) at various distances, *d*(*A,B*), and in various relative orientations, *rotX,Y,z*(*A,B*). The configuration of the *in vitro* (‘in’) reconstituted THO–UAP56 complex pair^28^ was not observed in endogenous TREX–mRNPs, due to the absence of an mRNA or mRNP substrate (see also panel **d**). **c.** Central atom distances and positions from TREX-A to -B (n=275) describe the surface of mRNP globules. The TREX-B central atom (THOC6 Glu 514 Χζ) is shown as a sphere and colored by its distance from the equivalent TREX-A atom. The dashed line indicates the pseudo-two-fold axis in TREX-A. The TREX-A (green) and its UAP56 (pink) is shown as ribbons. **d.** A t-SNE plot of TREX–mRNP pair distances and relative orientations revealed a lack of preferred TREX–TREX interaction modes (see Methods). Each TREX–mRNP pair is shown as a point, colored by the TREX-A to -B distance. We did not observe the *in vitro* THO–UAP56 pair (‘in’) or a parallel orientation of TREX pairs (‘p*’), which are both incompatible with TREX binding an mRNP. **e.** Examples of TREX–mRNPs containing three TREX complexes (A, B, and C) illustra how TREX can coat mRNP surfaces.

TREX–mRNPs vary in their dimensions and can include more than one TREX complex (Fig. 2b, Extended Data Fig. 4i). However, both mRNP details and additional TREX complexes blurred out during single particle averaging (Fig. 2c, Extended Data Fig. 5). To investigate how multiple TREX complexes can engage with mRNPs and to understand mRNP organization, we acquired 109 cryo-electron tomograms of the endogenous complexes. Tomogram reconstruction and denoising^49^ allowed us to directly visualize TREX–mRNP complexes (Extended Data Fig. 8). TREX–mRNPs vary in their volume and are roughly spherical, yet volume and sphericity were not correlated (Extended Data Fig. 9f, h, Methods). We picked individual TREX complexes by template matching for three-dimensional image classification and refinement, yielding a TREX density at 13 Å resolution (Extended Data Fig. 8a, b, f, Methods). The density showed an excellent fit to our single particle cryo-EM structure, including the UAP56 RecA1 lobe in monomers A and B (Extended Data Fig. 8a, b). To visualize TREX bound to diverse mRNPs, we overlaid the refined TREX complex coordinates from subtomogram averaging onto the denoised tomograms (Fig. 4a, Extended Data Fig. 8).

### TREX complexes coat the mRNP surface

TREX complexes bind exclusively at the mRNP surface and face the mRNP with its four UAP56 subunits (Fig. 4a, b, Extended Data Fig. 8, Supplementary Text 2). Binding of multiple TREX complexes to an mRNP might occur through TREX–TREX interactions, as observed for the recombinant TREX core THO–UAP56 complex (Extended Data Fig. 9j and ref.^28^). To assess this, we identified 275 TREX complex pairs, made up of TREX-A and -B, and aligned these on TREX-A (Methods). For each pair, we determined the relative TREX-A to -B orientations and distances, and plotted these in real-space (Fig. 4c, Extended Data Fig. 9a-e) or after dimensionality reduction (t-distributed Stochastic Neighbor Embedding, t-SNE)^50^ (Fig. 4d) Both approaches show that TREX pairs sample the mRNP surface randomly. Consistent with this, TREX-A to -B interactions occurred in only a fifth of TREX pairs (Extended Data Fig. 9i, j), suggesting that TREX–TREX contacts are not necessary for multiple complexes to bind one mRNP. The diverse TREX–mRNP architectures are also consistent with a lack of sequence- or position-specific TREX-binding sites in yeast or human mRNAs^13,51^. Our combined analyses indicate that TREX complexes associate independently of each other with mRNPs, which could be needed for TREX to accommodate the diversity of mRNP shapes and sizes.

Up to three TREX complexes can bind the same mRNP (Fig. 4a, e). In these particles, TREX coats the majority of the mRNP surface (Fig. 4a, e, Supplementary Video 6). We expect most mRNPs to contain multiple TREX complexes, since our conservative data analysis underestimates total TREX numbers (Supplementary Text 2, Methods). Based on our and published data^31,52^ and the estimated UBM concentration of 560 μM (Methods), we predict that two to three TREX complexes would bind to the average human mRNP that contains 3,500 mRNA nucleotides^19^.

### mRNPs form compact globules

The data also provides insights into the three-dimensional organization of nuclear mRNPs. mRNPs form compact but non-uniform globules that lack a rigid internal structure, consistent with RNAs adopting an ensemble of structural states^3^ (Figs 2c, 4a). With a median TREX–mRNP particle diameter of ~450 Å and an average mRNA length of 2.1 μm^19^, when fully extended, mRNAs within TREX–mRNPs would be compacted ~50-fold. We observe that mRNPs can be organized in layers (Figs 3a, 4), where proteins at the mRNP surface, such as the TREX complex, can serve regulatory roles, whereas proteins within the globule, such as SRSF family proteins, might serve organizational roles. Further, in an mRNP globule, the surface-to-volume ratio would be minimized.

**Extended Data Figure 10:**
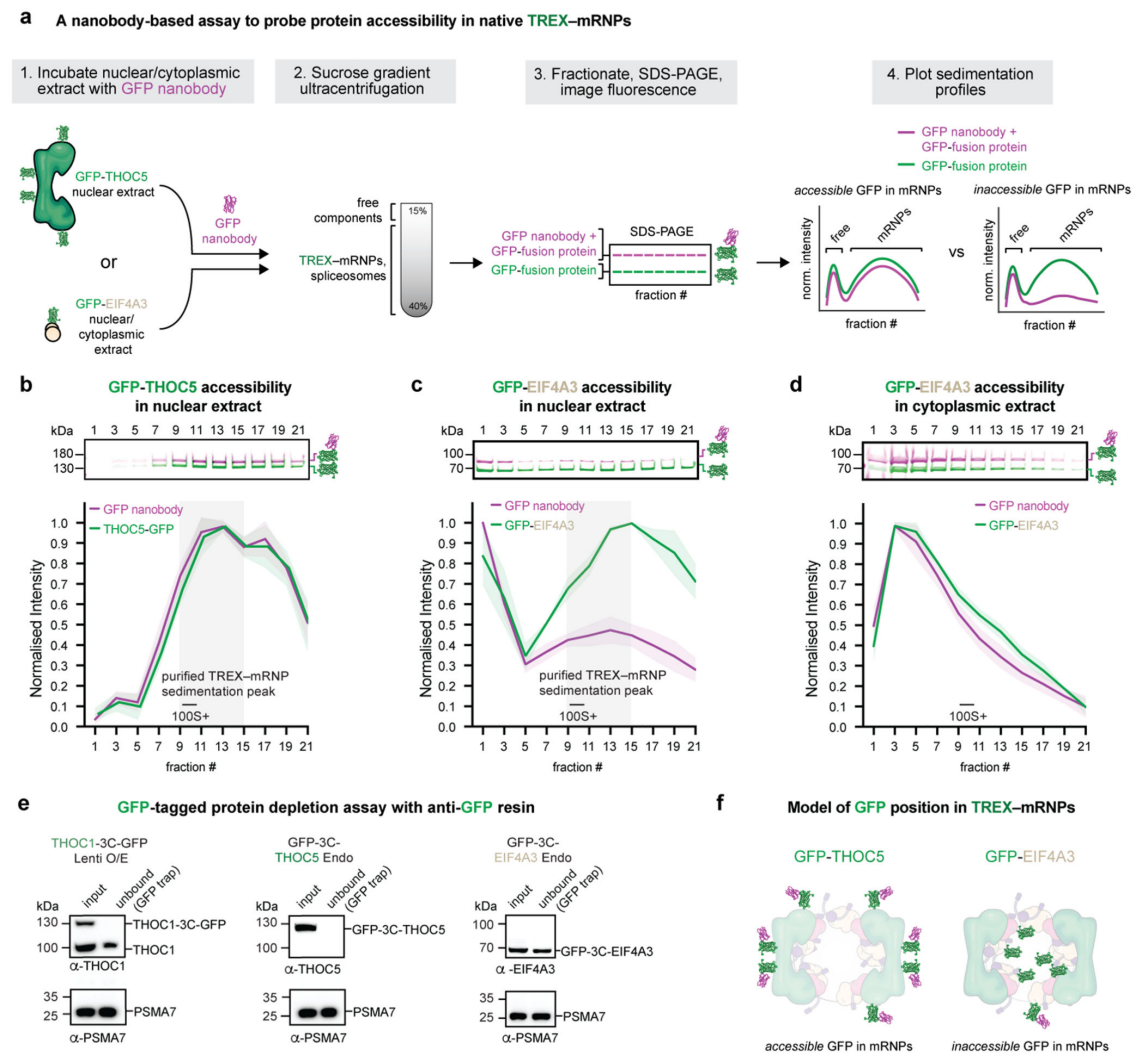
Probing protein accessibility in endogenous mRNP complexes. **a.** Schematic of the experiment to probe protein accessibility in mRNP complexes. The nuclear or cytoplasmic extract from K562 cells, tagged homozygously and endogenously with either GFP-3C-THOC5 or GFP-3C-EIF4A3, was incubated with a fluorescently labelled (AF647) 15 kDa anti-GFP nanobody. The extracts were then applied to a sucrose density gradient to separate free proteins from mRNPs, which migrate in heavy (later) sucrose gradient fractions. The gradient fractions were analyzed by SDS–PAGE. Due to high affinity of the anti-GFP nanobody to GFP (~1 pM), the nanobody stays bound to the GFP fusion during gel electrophoresis (see Methods for details). Fluorescence imaging allows quantification of the respective sedimentation profiles for the GFP fusion proteins (GFP-THOC5 or GFP-EI4A3, green channel) and the anti-GFP nanobody-bound fusion proteins (red channel, colored in magenta). When the GFP-tagged protein is accessible in mRNPs, then the anti-GFP nanobody signal closely follows the profile of the GFP-tagged protein. In contrast, when a GFP-tagged protein is inaccessible in mRNPs, the anti-GFP nanobody signal follows the GFP signal in early (light) sucrose gradient fractions that contain free proteins but shows reduced intensity in later (heavy) fractions. **b.** The anti-GFP nanobody signal closely follows the GFP-THOC5 signal, showing that GFP-THOC5 is accessible in mRNP complexes. Shown is the fluorescence signal from SDS-PAGE gels of GFP-THOC5 nuclear extract incubated with the AF647-labeled anti-GFP nanobody (top) and normalized sedimentation profiles (bottom). Sedimentation plots show mean normalized intensity values determined from three gels (solid lines) and standard deviations (transparent areas). The grey box indicates the peak gradient fractions of purified TREX–mRNPs (see Extended Data Fig. 4). This experiment was done four times. For gel source data, see Supplementary Figure 9. **c.** As for panel **b**, but for GFP-EIF4A3 in nuclear extract. In the high molecular weight fractions of the sucrose density gradient, GFP-3C-EIF4A3 is poorly accessible to the anti-GFP nanobody. This experiment was done four times. For gel source data, see Supplementary Figure 10. **d.** As for panel **b,** but for GFP-EIF4A3 in cytoplasmic extract. In the high molecular weight fractions of the sucrose density gradient, GFP-EIF4A3 remains accessible to the anti-GFP nanobody, in contrast to GFP-EIF4A3 in nuclear extract, which is shown in panel **c**. This experiment was done twice. For gel source data, see Supplementary Figure 11. **e.** Western blot experiment that shows the different depletion efficiencies of THOC1-GFP (ectopically overexpressed; Lenti O/E), GFP-THOC5 (endogenously tagged; endo), or GFP-EIF4A3 (endogenously tagged; endo) from nuclear extract using GFP-Trap resin (containing an anti-GFP nanobody coupled to 90 μm agarose beads) after three rounds of depletion. While THOC1-GFP and GFP-THOC5 are completely depleted in the supernatant, GFP-EIF4A3 is very inefficiently depleted. Anti-PSMA7 blots (a proteasome subunit) serve as loading controls. These experiments were done three times. For gel source data, see Supplementary Figure 12. **f.** Cartoon model showing the position and nanobody-accessibility of GFP-tagged THOC5 or EIF4A3 in TREX–mRNPs, based on the accessibility to the anti-GFP nanobody and anti-GFP resin in panels **e** and **f**.

The TREX–mRNP architecture predicts that mRNPs should be less accessible to the environment than TREX. To test this, we established an assay to probe for accessibility of either the TREX subunit THOC5 or the EJC subunit EIF4A3 in native TREX–mRNPs (Extended Data Fig. 10a). We incubated nuclear extracts from either CRISPR-Cas9 knock-in GFP-THOC5 or GFP-EIF4A3 K562 cells with an anti-GFP nanobody, carrying a fluorescent label (AF647). We then applied these nuclear extract-nanobody mixtures to sucrose gradient ultracentrifugation to investigate if the nanobody would bind the GFP-fusion protein in the heavy gradient fractions, where TREX–mRNPs migrate, or in the light gradient fractions, where free proteins migrate. The nanobody binds GFP-THOC5 well across all fractions (Extended Data Fig. 10b), consistent with TREX residing at the mRNP surface. In contrast, the nanobody binds GFP-EIF4A3 poorly in the heavy gradient fractions (Extended Data Fig. 10c), consistent with a reduced accessibility of mRNP-bound EIF4A3 to the solvent. Notably, in cytoplasmic extract, the nanobody could bind GFP-EIF4A3 also in the heavy gradient fractions (Extended Data Fig. 10d), in agreement with a lower degree of compaction in cytoplasmic compared to nuclear mRNPs and the absence of TREX in the cytoplasm^15,16^. Further, we observed that all endogenously tagged GFP-THOC5 or lentiviral overexpressed THOC1-GFP could be immunoprecipitated using anti-GFP nanobody beads from the respective nuclear extracts (Extended Data Fig. 10e), whereas GFP-EIF4A3 depletion was inefficient. Together, these data support the observed TREX–mRNP architecture with TREX binding at the surface of mRNP globules that contain the EJC at their center.

### Model for mRNA packaging and export

**Figure 5:**
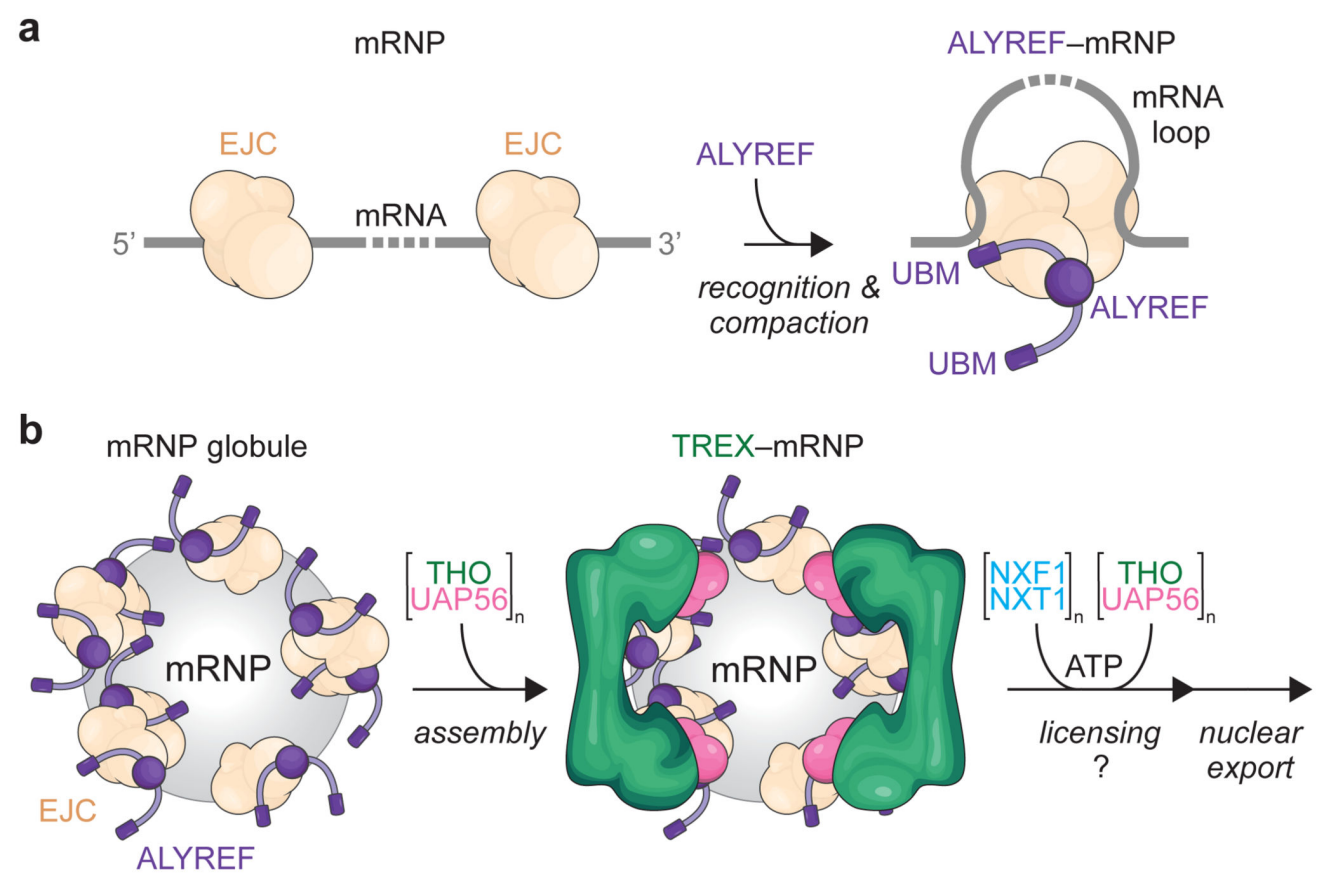
Model for mRNA packaging. **a.** The TREX subunit ALYREF recognizes and may compact mature mRNPs by bringin neighboring EJCs together through multivalent protein-protein and protein-mRNA interactions. **b.** Compacted ALYREF-mRNPs may form mRNP globules containing a high concentration of ALYREF UBMs at the mRNP surface, where TREX complexes subsequently assemble. TREX licenses loading of the mRNA export factor, NXF1–NXT1, onto mRNPs and this may require an ATP-dependent step (see main text for details). mRNA export factor loading may occur in the nucleoplasm^59^ or at the nuclear pore complex^60,61^ and thereby license mRNPs for nuclear export.

Our results suggest a model for TREX-dependent nuclear mRNA packaging and export across mRNA scales (Fig. 5). Locally, multiple ALYREF molecules could specifically recognize spliced mRNPs through multivalent interactions with the CBC or EJCs, leading to a high local UBM concentration at the mRNP surface. Globally, ALYREF–mRNPs may form compact globules, which specifically assemble multiple TREX complexes on their surface. This would allow for UAP56 to engage with the mRNA and to promote loading of the mRNA export factor onto mature mRNPs.

During these events, TREX may use four mechanisms to promote mRNA biogenesis. First, mRNP-coating by TREX would spatially confine mRNPs and promote efficient and specific mRNA packaging and export-licensing through multivalent interactions^28^. The combined multivalent binding of THO^28,41^ and of ALYREF^53^ to UAP56 may jointly stimulate the UAP56 ATPase activity and mRNA binding. Since UAP56 is not yet clamped onto mRNA in TREX–mRNPs (Fig. 2d, Extended Data Fig. 5j), this ATP-dependent step may regulate a subsequent mRNP remodeling and mRNA export factor loading (Fig. 5). Second, the TREX–mRNP organization could explain how the THO complex releases from mRNPs prior to nuclear export, due to its location at the mRNP surface. Third, sequestration of mRNA from chromatin and the nucleoplasm would prevent harmful RNA–DNA interactions during transcription (R-loops) or RNA–RNA contacts during mRNP maturation. This could explain how defects in TREX and other mRNA-processing proteins cause genome instability^14^, indirectly, through the accumulation of loose mRNA near DNA. The absence of TREX subunits at R-loops is consistent with this model^54^. Fourth, coating by TREX may explain how mRNPs are protected from nuclear mRNA degradation machineries^55,56^. Future work will be needed to probe each mechanism in detail.

For mRNP export specifically, mature mRNPs may more efficiently diffuse through the crowded nucleoplasm^57^ and the nuclear pore complex, due to the globule architecture^58^. Analogous to TREX, the mRNA export factor may be loaded onto the mRNP surface, either in the nucleoplasm^59^ or at the nuclear pore complex^60,61^, and would thereby solubilize mRNPs for transport through the hydrophobic barrier of the nuclear pore complex.

At both local and global mRNP scales, we identify a unifying mechanism for nuclear mRNP recognition and packaging, which depends on multivalent and low-affinity interactions. Both rely on specific protein-protein and non-specific protein-mRNA interactions, which can explain how mature mRNPs are identified without discriminating among mRNAs that differ in length and sequence. Finally, both interaction types may shape nuclear mRNPs into globules. mRNPs thereby form a unique molecular surface that can be functionalized to regulate nuclear mRNA expression, maintain mRNA fluidity in the nucleoplasm^57^, and promote mRNA nuclear export.

## Methods

### Vectors and sequences

The human ALYREF, EIF4A3, UAP56 and CASC3_SELOR_ ORFs were respectively cloned into a pOPINB vector for expression in *E. coli.* The tags in all constructs can be cleaved by digestion with 3C PreScission protease. ALYREF_FL_ was cloned with an N-terminal Maltose-binding protein (MBP) fusion and a C-terminal 6x-histidine (His) tag.

ALYREF_N_, ALYREF_55-182_, and ALYREF_RRM_ contained an N-terminal 10x-His tag fused to MBP. ALYREF_NARRM_ and CASC3_SELOR_ contained an N-terminal 6x-His-MBP tag. ALYREF_C_ contained an N-terminal 10x-His and a C-terminal MBP tag. Full-length EIF4A3 was cloned with N-terminal 6x-His tag and UAP56 with an N-terminal 10x-His tag fused to Twin-Strep or MBP. The plasmid for 6x-His-TEV-Y14_(66-154)_–MAGOH dimer expression in *E. coli* was a gift from Sebastian Falk, Max Perutz Labs, Vienna. The human 6-subunits of the THO complex were cloned into a modified pACEBact vector (Geneva Biotech) for insect cell expression as previously described in^28^. Sequences are available upon request.

### Protein Purification

#### ALYREF

10x-His-MBP-3C-ALYREF_N_ was expressed in E. coli BL21 DE3 RIL cells grown in LB media induced at OD600 of 1.0 with 0.5 mM IPTG and incubated at 37°C for 3 h. Bacteria were lysed by sonication in buffer A (50 mM HEPES, pH 7.9, 500 mM NaCl, 5% (v/v) Glycerol, 20 mM Imidazole, 0.5 mM EDTA, cOmplete EDTA-free protease inhibitor cocktail). The clarified supernatant was filtered through 0.45 μm filters and loaded on a HisTrap HP 5ml column, equilibrated in buffer B (50 mM HEPES, pH 7.9, 500 mM NaCl, 5% (v/v) Glycerol, 20 mM Imidazole, 1mM DTT). The column was washed with buffer B and then eluted with a linear gradient from 20 mM to 300 mM Imidazole. 10x-His-MBP-ALYREF_N_ was diluted in buffer C (25 mM HEPES, pH 7.9 5% (v/v) Glycerol, 1 mM DTT) to 200 mM NaCl and then further purified by cation exchange chromatography using a HiTrapSP HP 5ml column, equilibrated in buffer C with 200 mM NaCl. After washing, the protein was eluted with a linear gradient from 200 mM to 1M NaCl, peak fractions were concentrated and loaded on a HiLoad 16/600 Superdex 75 pg column, equilibrated in buffer D (25 mM HEPES, pH 7.9, 400 mM NaCl, 5% (v/v) Glycerol, 1 mM DTT). The purified protein was concentrated to 15mg/ml, flash frozen and stored at -80 °C. All other ALYREF constructs (ALYREF_FL_, ALYREF55-182 and interface mutants, ALYREF_NΔRRM_, ALYREF_C_ and ALYREF_RRM_) were purified using a similar strategy to 10x-His-MBP-3C-ALYREF_N_with the following exceptions: 10x-His-MBP-3C-ALYREF_RRM_ and 10x-His-MBP-3C-ALYREF_55-182_ interface mutants did not require the ion exchange step as the 260/280 ratio was below 0.6, while 10x-His-MBP-3C-ALYREF_55-182_ required an anion exchange step with a HiTrapQ column. For ALYREF_55-182_ and interface mutants, ALYREFN_ARRM_, ALYREF_C_ and ALYREF_RRM_ the final gel filtration salt concentration was lowered to 250 mM NaCl. To obtain untagged ALYREF_N_, the 10x-His-MBP tag was cleaved by 3C PreScission protease after the first affinity step with the HisTrap HP at 4 °C under light agitation. The cleaved tag and the un-cleaved protein were removed using a reverse affinity chromatography by loading the sample on a HisTrap HP 5ml column equilibrated in buffer C with 300 mM NaCl. The flowthrough from the previous step was then further purified by following the steps as with 10x-His-MBP-ALYREF_N_.Except for the initial salt concentration before cation exchange chromatography, this was kept at 300 mM NaCl throughout.

#### EIF4A3

We expressed 6x-His-3C-EIF4A3 in E. coli BL21 DE3 RIL cells grown in LB media at 37°C and induced at a OD600 of 1.0 with 0.5 mM IPTG. Cells were harvest after 3 h of induction. Sonication was performed as described for ALYREF constructs. The supernatant was clarified using centrifugation, filtered through 0,45 μm filters and applied to a HisTrap HP 5ml column pre-equilibrated in buffer B. The column was washed with 40 mM imidazole and then eluted with a step gradient to 300 mM imidazole. Peak fractions were diluted with buffer C to 100 mM NaCl and loaded on a HiTrapQ HP 5ml column, equilibrated with buffer C containing 100 mM NaCl. After washing, the protein was eluted with a linear gradient to 800 mM NaCl, peak fractions were concentrated and loaded on a HiLoad 16/600 Superdex 75 pg, equilibrated in buffer F (25 mM HEPES, pH 7.9, 150 mM NaCl, 10% (v/v) Glycerol, 1 mM DTT). The purified protein was flash frozen with a concentration of 1mg/ml and stored at -80°C.

#### MAGOH–Y14

6x-His-TEV-Y14(66-154) was co-expressed with full length MAGOH in E. coli BL21 DE3 RIL cells grown at 37°C for 3 hours in complete TB media until reaching an OD600 of 2.0. After a 30 min incubation at 4 °C, the bacteria were further cultured at 18 °C until reaching an OD 600 of 4. Then, cells were induced with 0.4 mM IPTG and incubated at 18 °C for 12 h. The harvested bacteria were lysed by sonication in Buffer G (20 mM TRIS pH 7.5, 50 mM NaH_2_PO_4_ pH 8, 300 mM NaCl, 10% (v/v) Glycerol, 20 mM Imidazole, 1 mM DTT, 0.1 mM PMSF, and 0.2 U/mL DNase I, cOmplete EDTA-free protease inhibitor cocktail). The clarified supernatant was loaded to a HisTrap HP 5ml column equilibrated in buffer G without PMSF and DNase. After washing with this buffer, the MAGOH-Y14 dimer was eluted using a step gradient to 300mM imidazole. Peak fractions were pooled, and the His-tag was cleaved using TEV protease during a 12 h dialysis at 4 °C against buffer H (20 mM TRIS pH 7.5, 300 mM NaCl, 5% (v/v) Glycerol, 20 mM imidazole, 1 mM DTT). The cleaved tag and the uncleaved protein were removed using a reverse affinity chromatography by loading the sample on a HisTrap HP 5ml column equilibrated in buffer G without PMSF and DNase. The flowthrough from the previous step was then applied to a HiLoad 16/600 Superdex 75 pg column equilibrated in buffer I (25 mM HEPES, pH 7.9, 300 mM NaCl, 5% (v/v) Glycerol, 1 mM DTT). Peak fractions were pooled and concentrated to

#### THO complex

Recombinant THO complex (THOC1, -2 residues 1-1203, -3, -5, -6, -7) was purified as previously described in ref.^28^.

To generate a monomeric THO construct (THO_Monomer_), we modified the construct described in ref.^28^ by excluding THOC6 (tetramer interface) and truncating THOC5 and THOC7 parallel coiled coils (THOC5 1-224, THOC7 1-159; dimer interface). Then, we expressed this new construct in Hi5 insect cells using baculovirus. Insect cell pellets were resuspended in buffer A and lysed by sonication. The lysate was cleared by centrifugation (first for 30 min at 18,500 rpm, then for 1 h at 40,000 rpm in a Ti45 rotor). The supernatant was filtered through 0.45 μm filters and applied to a HisTrap HP 5 ml column, previously equilibrated in buffer B. The column was washed with buffer B and eluted with a linear gradient from 20 mM to 100 mM imidazole. Peak fractions were pooled, diluted 1:5 with buffer C and applied to a HiTrap Q ion exchange column for further purification that was equilibrated in buffer C containing 150 mM NaCl. The column was washed with this buffer and the protein was eluted with a linear gradient from 150 mM to 500mM NaCl. Finally, peak fractions were further purified via gel filtration on a HiLoad 200 16-60 column equilibrated in buffer D, containing 250 mM NaCl. The purified protein was concentrated to 8 mg/mL and flash frozen in liquid nitrogen. Gel filtration confirmed that the construct was monomeric in solution.

#### UAP56

Full length human 6x-His-TwinSTREPII-3C-UAP56, 6x-His-MBP-3C-UAP56 and 6x-His-MBP-3C-UAP56 RQRK mutant (UBM-binding interface mutant, R208S Q212A R216S K241S) were purified as previously described in ref.^28^ except the tags were not cleaved during purification.

#### CASC3_SELOR_

6x-His-MBP-3C-CASC3_SELOR_was expressed in E. coli BL21 DE3 RIL cells grown in LB media induced at OD600 of 1.0 with 0.5 mM IPTG and incubated at 37°C for 3 h. Bacteria were lysed by sonication in buffer A containing 300mM NaCl. The clarified supernatant was filtered through 0.45 μm filters and loaded on a HisTrap HP 5ml column, equilibrated in buffer B containing 300mM NaCl. The column was washed with buffer B, 300 mM NaCl, 40mM Immidazole and then eluted with a step gradient from 40 mM to 300 mM Imidazole. 6x-His-MBP-3C-CASC3_SELOR_ was diluted with buffer C to 100 mM NaCl and then further purified by anion exchange chromatography using a HiTrapQ HP 5ml column, equilibrated in buffer C with 100 mM NaCl. The protein was eluted with a linear gradient from 200 mM to 800 mM NaCl, peak fractions were concentrated and loaded on a HiLoad 16/600 Superdex 75 pg column, equilibrated in buffer D, containing 150 mM NaCl. The purified protein was concentrated to 6mg/ml, flash frozen and stored at -80°C.

### Pulldowns and gradients with recombinant protein

#### ALYREF_N_–EJC–RNA reconstitution

To reconstitute the 10xHis-MBP-3C-ALYREF_N_–EJC–RNA complex, 10xHis-MBP-3C-ALYREF_N_was mixed with a two-fold molar excess of 6His-3C-EIF4A3 and Y14 (residues 66-154)–MAGOH dimer and a fourfold molar excess of a 15 nucleotide (nt) single stranded (ss) poly-U RNA in binding buffer (25 mM HEPES, pH 7.9, 50 mM NaCl, 5 mM MgCl_2_, 10% (v/v) Glycerol, 1 mM TCEP, 1 mM AMP-PHP) and the sample was incubated overnight under rotation at 4 °C.

#### EJC reconstitution assay (pulldown)

The assembled 10xHis-MBP-3C-ALYREF_N_–EJC–RNA complex was immobilized on HighFlow Amylose resin (NEB) equilibrated with equilibration buffer (25 mM HEPES, pH 7.9, 50 mM NaCl, 5 mM MgCl_2_, 10% (v/v) Glycerol, 1 mM TCEP, 0.05% (v/v) Igepal). After washing with equilibration buffer, the complex was eluted using equilibration buffer supplemented with 15 mM Maltose. Complex assembly was monitored using SOS-PAGE stained with Coomassie blue.

#### ALYREF_N_–EJC–RNA multimerization assay

10x-His-MBP-3C-ALYREF_N_–EJC–RNA complex was assembled as described above except the complex was additionally assembled either on a 50 nt ssRNA derived from the Adenovirus Major Late (AdmL) pre-mRNA^33^ or a 15 nt ssRNA poly-Uridine RNA to assess the influence of RNA length on multimerization. After pulldown on MBP and maltose elution, the samples were loaded on a 15-40% sucrose density gradient and centrifuged at 50,000 rpm for 16 hrs in a SW60 Ti rotor (Beckman Coulter). We collected fractions and loaded every second one on an SOS-PAGE and stained the gel with Coomassie blue. We quantified the band intensity of MAGOH across the sedimentation profile of ALYREF–EJC–RNA complexes in ImageJ^70^ and then plotted the normalized intensities. The sedimentation coefficients of multimeric states were simulated using the *CowSuite* software (https://www.cow-em.de), considering that one 10x-His-MBP-3C-ALYREF_N_–EJC–RNA complex is ~150kDa. Predicted sedimentation coefficients for monomers, dimers, trimers, tetramers, pentamers and hexamers are 7S, 11S, 15S, 18S, 21S and 24S, respectively.

#### EJC reconstitution assay with ALYREF truncations

The complexes were assembled and the EJC reconstitution was assayed as described in *ALYREF_N_–EJC–RNA reconstitution* and *EJC reconstitution assay: pulldown*, except different ALYREF truncations were used, as indicated in Extended Data Fig.1d.

#### ALYREF55-182–EJC–RNA multimerization assay with nuclease digestion

10x-His-MBP-3C-ALYREF55-182–EJC–RNA complexes were assembled as described in ALYREF_N_–EJC–RNA reconstitution on 15nt single-stranded poly-U RNA. After overnight assembly, a final concentration of 20μg/ml benzonase was added to one of two samples. Digestion was carried out for 4 hrs at 4 °C, on a rotating wheel. After pulldown on MBP, the maltose elutions were loaded on a 15-40% sucrose density gradient and centrifuged at 45,000 rpm for 16 hrs in a SW60 Ti rotor. We collected fractions and monitored sedimentation profiles as described for the ALYREF_N_–EJC–RNA multimerization assay.

#### EJC reconstitution assay with ALYREF mutants in ALYREF–EJC interface

The complexes were assembled and the EJC reconstitution was assayed as described in *ALYREF_N_–EJC–RNA reconstitution* and *EJC reconstitution assay: pulldown*, except different ALYREF mutants we used, as indicated in Extended Data Figs. 1g, h.

#### ALYREF_55-182_–EJC–RNA multimerization assay with interface mutants

The complexes were assembled on 15nt single-stranded poly-U RNA as described above, except different ALYREF interface mutants or 6x-His-MBP-3C-CASC3_SELOR_ were used as indicated in Extended Data Fig. 1. After pulldown on MBP, the maltose elutions were loaded on a 15-40 % sucrose density gradient and centrifuged at 25,000 rpm for 16 hrs in a SW60 Ti rotor. We collected fractions and monitored sedimentation profiles by as described in ALYREF_N_–EJC–RNA multimerization assay.

#### THO–UAP56 and ALYREF interaction assay

The recombinant THO complex was incubated with two-fold molar excess of UAP56 for 10 min at room temperature and either directly applied to sucrose density gradients or first incubated with five-fold molar excess of untagged ALYREF_N_ for 30 min at room temperature. Samples were applied to 15-40 % sucrose gradients and centrifuged in a SW60 Ti rotor for 16 hrs at 32,000 rpm. Every second fraction was analyzed on SDS-PAGE and stained with Coomassie blue. The band intensities of TH0C2 and ALYREF were quantified in ImageJ^70^ and the normalized intensities were plotted.

#### TREX–EJC–RNA reconstitution

10x-His-MBP-3C-ALYREF_N_–EJC–RNA complex was assembled as described on a 50 nt ssRNA. Instead of eluting with maltose, the immobilized 10x-His-MBP-3C-ALYREF_N_–EJC–RNA complex was incubated at RT for 30 min with equimolar 6x-His-TwinSTREPII-3C-UAP56 supplemented with 0.05 mM ATPγS and 3C PreScission protease (which was previously incubated for 15 min at RT). Then, equimolar amounts of THO complex were added to the sample and further incubated for 30 min at RT and additional 30 min at 4 °C. The amylose resin was sedimented by centrifugation and the eluted TREX–EJC–RNA assembly was collected from the beads.

#### Monitoring TREX–EJC–RNA assembly

Eluted TREX–EJC–RNA complexes were loaded on a 15%-40% sucrose density gradient and centrifuged at 20,000 rpm for 16 hrs in a SW60 Ti rotor. We collected fractions and analyzed every other fraction by SDS-PAGE stained with Coomassie blue. The sedimentation coefficients of multimeric states were simulated using the *CowSuite* software.

#### TREX–EJC–RNA reconstitution assay

ALYREF_N_–EJC–RNA and ALYREF_55-182_–EJC–RNA were assembled as described above on a 15 nt ssRNA. Then, THO–UAP56 (Extended Data Fig. 1m) or THO_monomer_–UAP56 (Extended Data Fig. 1n) was added as described in *TREX–EJC–RNA reconstitution*, but without ATPγS. The eluates were loaded on a 15%-40% sucrose density gradient and centrifuged at 23,000 rpm for 16 hrs in a SW60 Ti rotor. We collected fractions and analyzed every other fraction by SDS-PAGE stained with Coomassie blue. The band intensity of reference subunits was quantified in ImageJ^70^ and the normalized intensities were plotted. THOC2 was used as a THO–UAP56 marker and MAGOH was used as a marker for the EJC. The normalized intensities of MAGOH were multiplied by a factor of 3 for better visualization.

#### RNA affinity measurements

RNA affinities were determined using a filter binding assay. Proteins were incubated for 45 min at 20°C in presence of 50 nM fluorescein-labelled 400 nucleotide RNA derived from the Adenovirus major late pre-mRNA in binding buffer (150 mM KCl, 20 mM HEPES, pH 7.9, 2 mM MgCl_2_, 5% (v/v) Glycerol, 0.5 mM TCEP, with or without 1 mM ATPγS); protein concentrations ranged from 1.2 nM to 5 μM. After incubation, 6 μL of each sample were applied under vacuum to a stack of a nitrocellulose membrane (Amersham™ Protran™ NC, CAS 10600002) and an Amersham Hybond N+ membrane (CAS RPN303B) and wells were washed with 100 qL binding buffer (without RNA). Membranes were then separated, and the fluorescein signal was imaged on a laser scanner (Sapphire scanner, Azure BioSystems) with a pixel size of 100 μm at a wavelength of 488 nm. Each sample was measured in two independent experiments, with three technical replicates per experiment. Fluorescent intensities on both membranes were measured using a circular ROI in Fiji^70^, and the membrane background signal was subtracted from each point. The amount of bound RNA was calculated by dividing the fluorescent signal found on the nitrocellulose membrane by the total signal (signal on the nitrocellulose membrane plus signal on the hybond membrane). Binding affinities were calculated using the “Specific binding with Hill-slope” function in GraphPad Prism, with Bmax constrained to 1. The function has the form of Y=Bmax*X^h^/(KD^h^ + X^h^), where Y is the fraction of bound RNA, X the protein concentration, h the hill coefficient, and Bmax the observed maximum binding.

### Cell lines

#### Generation of cell line overexpressing THOC1-3C-GFP

The production of the cell line was previously described in ref ^28^. Generation of cell lines overexpressing FLAG-ALYREF. Wildtype ALYREF or mutant ALYREF^M-*c*+Δ*d*^ cDNA and a N-terminal 3xFlag tag were cloned into a lentiviral vector backbone (Addgene plasmid #31485), yielding a plasmid containing pRRL-SFFV 3x-Flag-Aly/REF-p2a-mCherry. For an overexpression of ALYREF variants in K562 THOC1-GFP overexpressing cells, lentiviral particles carrying the 3x-Flag-ALYREF constructs were generated using Lenti-X cells (Takara) by polyethylenimine transfection (Polysciences) of the viral plasmid and helper plasmids pCMVR8.74 (Addgene plasmid #22036) and pCMV-VSV-G (Addgene plasmid #8454), according to standard procedures. THOC1-GFP overexpressing K562 (DSMZ) cells were infected at limiting dilutions and GFP and mCherry double positive cells were isolated using a BD FACSAria III cell sorter (BD Biosciences). Viral integration was confirmed by immunoblotting for ALYREF (antibodies: Ancam ab202894 and Proteintech 16690-1-AP) and FLAG (A8592; Sigma-Aldrich).

#### Generation of a knock-in cell line expressing endogenous GFP-3C-THOC5

K562 cells (DSMZ) were edited to express a EGFP-THOC5 fusion protein using a modification of a previously described CRISPR/Cas9 knock-in protocol^71^. In short, the gRNA was designed using the Benchling.com CRISPR gRNA design tool (Benchling; aaacTGTCATCAGAATCGAGCAAAC) and cloned into the plasmid pLCG (hU6-sgRNA-EFSSpCas9-P2A-mCherry)^72^, a gift from J Zuber, IMP, Vienna. The 500 bp sequences flanking the THOC5 start codon were obtained by PCR on genomic DNA obtained from K562 cells and subcloned into pLPG vector, a gift from J. Zuber, digested with MluI using Gibson Assembly (NEB), yielding the final vector pLPG-GFP-AID (5’ BlastR-P2A-eGFP-AID-3C). K562 cells were grown in RPMI medium supplemented with 10% FBS (Sigma), 2% L-Glutamine (Gibco), 1% Sodium Pyruvate (Sigma) and 1% Penicillin Streptomycin (Sigma) and transfected with the HDR donor and the Cas9 plasmids using the Neon electroporation device (Invitrogen) according to the user manual (for suspension cells). 14 days post-transfection, after several passages and Blasticidin selection (10μg/ml, Invitrogen), cells were subjected to fluorescence activated cell sorting (FACS) using a BD FACSAria III (BD Biosciences). Cells expressing the EGFP-tag were sorted into 96 well plates. After allowing single cells to regrow for approximately two weeks, clones with homogeneous GFP fluorescence were genotyped (primers: AGCAGGGGAAAAGACATGGA, CTTGAGCCCAGGAAATGCAG). For homozygously edited cells, expression of GFP-THOC5 was analyzed by western blotting with anti-THOC5 (ab137051; Abcam) and anti-GFP antibodies (A11122; Invitrogen).

#### Generation of a knock-in cell line expressing endogenous GFP-3C-EIF4A3

GFP-3C-EIF4A3 K562 cells were generated using an identical N-terminal CRISPR-Cas9 tagging strategy as described for GFP-3C-THOC1. The gRNA sequence was aaacACTCTGAATCATGGCGACCA) and the 500bp sequences flanking the EIF4A3 start codon were obtained by PCR on genomic K562 DNA. Colonies were genotyped using PCR primers (GCAAACGGTGAAGACACACC and CAAAACCCGTAAAGGCGCAA). Homozygous clones were further analyzed by western blotting to validate the homozygous knock-in of the tag using anti-EIF4A3 (ab180519; Abcam) and anti-GFP antibodies (A11122; Invitrogen).

#### Preparation of nuclear extract (NE)

Nuclear salt wash extract was prepared from a K562 cell line overexpressing THOC1-3C-AID-GFP as previously described in ref.^28^.

#### Rapid cell fractionation (RCF) nuclear extract

The protocol was adapted from the protocol by Suzuki et. al.,^65^ for preparative scales. 1L of confluent overexpressing THOC1-3C-AID-GFP K562 cells were harvested and resuspended in REAP Buffer (0.1% Igepal, 100 mM KCl, 25 mM HEPES, pH 7.9, 1mM DTT) and spun at 3,000 rcf for 3.5 min, the cytosolic fraction was removed by inversion. The nuclear pellet was washed with REAP buffer and spun at 1,500 rcf for 2 min, then it was washed once again with REAP buffer lacking Igepal but containing 5% glycerol and finally resuspended in that same buffer. The obtained nuclei were processed by cryo-milling and freshly thawed for use and spun on a bench top centrifuge for 15 min at 8,000 rcf.

### Purification and characterization of endogenous material

#### ALYREF interface mutant interaction assay in RNase-treated nuclear extract

NE from overexpressing wild-type FLAG-ALYREF or FLAG-ALYREF^M-*c*+Δ*d*^ was prepared as described. 250 μl of each NE was supplemented a final concentration of 5 mM MgCl_2_, cOmplete EDTA-free protease inhibitor cocktail and digested with 30 μg benzonase per ml of NE for 16 hrs at 4°C. The extracts were incubated for 4 hrs at 4°C with anti-FLAG M2 previously equilibrated with Buffer A (25 mM HEPES, pH 7.9, 150 mM NaCL, 5 mM MgCl_2_, 0.5% Triton X-100 and 10% Glycerol, 0.5 mM TCEP). After washing, samples were eluted by boiling and interaction in nuclear extracts with mRNP-associated factors was analysed by western blotting following standard protocols. We used horseradish peroxidase (HRP)-coupled α-FLAG (CAS 86861S or A8592-.2MG, Cell Signalling technology or Sigma Aldrich, diluted 1:2000), α-EIF4A3 (CAS AB180573 or AB180519, Abcam, diluted 1:500) and α-NCBP1 (CAS 16856683, Proteintech, diluted 1:1000). Primary antibodies were incubated overnight at 4 °C. For detection we used a secondary goat-anti-rabbit antibody coupled to horseradish peroxidase (CAS 31466) (except for HRP-coupled α–FLAG) and developed the membrane with Pierce ECL reagent (CAS 32106).

#### TREX–mRNP sedimentation coefficient determination

THOC1-3C-AID-GFP K562 NE was treated with 1 μg nuclease (benzonase) per mL NE and simultaneously incubated with GFP-Trap Agarose resin (Chromotek) pre-equilibrated with binding buffer (20 mM HEPES, pH 7.9, 100 mM KCl, 2 mM MgCl_2_, 8% (v/v) Glycerol, 0.05 % (v/v) Igepal CA-630, 1 mM DTT, protease inhibitor cocktail) for 12 hrs at 4°C. After washing, the TREX–mRNP complexes were eluted by cleavage using 3C PreScission Protease diluted in binding buffer for 2 hrs. The eluate was loaded onto a 15–40% w/v sucrose density gradient and spun at 15,000 rpm for 16 hrs in a SW60 Ti rotor. Fractions were collected and analyzed with SDS-PAGE stained with Coomassie blue. The sedimentation coefficients were simulated using the CowSuite software. Individual bands were identified using Mass spectrometry.

#### Mass spectrometry analysis of TREX–mRNP complexes

Samples were prepared as described above in ‘*TREX–mRNP sedimentation coefficient determination*’, except the peak TREX–mRNP fractions were pooled and buffer exchanged in a buffer containing 25 mM HEPES, pH 7.9, 100 mM KCl, 2 mM MgCl_2_ and 1 mM TCEP using a spin column for subsequent in solution protein identification using mass spectrometry analysis.

#### Western blot for NXF1 in TREX–mRNPs

We performed THOC1-GFP IPs as before and analyzed nuclear extract (input) and eluted fractions using western blotting for α-THOC1 (Sigma, HPA019096, diluted 1:1000), α-NXF1 (ab129160, diluted 1:2000), α-EIF4A3 (ab180519, 1:1000) and α-PSMA7 (PA5-21379, diluted 1:2000). Primary antibodies were incubated overnight at 4 °C. For detection we used a secondary goat-anti-rabbit antibody coupled to horseradish peroxidase (CAS 31466) and developed the membrane with Pierce ECL reagent (CAS 32106).

#### SRSF1-phosphorylation assay of TREX–mRNP complexes

We performed THOC1-GFP IPs as before and analyzed the eluted fractions using western blotting. Samples were either analyzed directly or first de-phosphorylated with lambda-phosphatase (NEB) according to manufactures instructions. For western blotting, we used α-THOC1 (CAS HPA019096, diluted 1:1000) α-SRSF1 (CAS PA5-30220, diluted 1:2000). Primary antibodies were incubated overnight at 4 °C. For detection we used a secondary goat-anti-rabbit antibody coupled to horseradish peroxidase (CAS 31466) and developed the membrane with Pierce ECL reagent (CAS 32106).

#### TREX–mRNP RNA extraction and sequencing

After purification of endogenous mRNPs by pulldown on THOC1-GFP as described above, RNA was isolated from mRNPs by phenol-chloroform extraction and ethanol precipitation. Extracted RNA was treated with Invitrogen TURBO DNA-free kit (Thermo Fisher Scientific). 500 ng RNA were used to generate 3’-end sequencing libraries with a commercially available kit (QuantSeq 3’ mRNA-Seq Library Prep Kit FWD for Illumina). Libraries were sequenced as spike-ins using MiSeq SR150 on MiSeq2.

#### Analysis of QuantSeq data

Gene and 3’UTR annotations were obtained from the UCSC table browser (https://genome.ucsc.edu/cgi-bin/hgTables, April 2020) and refined utilizing 3’GAMES (https://github.com/poojabhat1690/3-GAmES). Adapters were trimmed from sequencing reads using cutadapt through the trim_galore (version 0.6.0) tool with adaptor overlaps set for 3 bp for trimming. Trimmed reads were further processed using SlamDunk v0.3.4 (http://t-neumann.github.io/slamdunk/), running the full analysis pipeline (slamdunk all), running plain Quantseq alignment without SLAM-seq scoring, aligning against the human genome (hg38) with the above described 3’UTR annotations, reporting up to 100 alignments for multi-mappers, activating multi-mapper retention strategy, filtering for variants (variant fraction of 0.2) and filtering for base quality cutoff >27. Other parameters were left to default settings. The analysis was restricted to genes with >1 read across all 3 replicates and gene type annotations were retrieved from biomaRt (April 2022) to determine the gene-type representation in endogenous TREX–mRNPs.

#### GFP-tagged protein depletion assay from nuclear extract using GFP-trap resin

200 μl of NE (THOC1-3C-GFP, GFP-3C-THOC5 or GFP-3C-EIF3A4), were depleted three times with 75 μl of GFP-trap resin for 4 hrs each. For western blot analysis, the concentration of input NE and of the last unbound fraction were normalized using absorbance at 280 nm. Western blotting was described as above, with antibodies for THOC1 (Sigma, HPA019096, diluted 1:1000) or THOC5 (ab228694, diluted 1:1000), EIF4A3 (ab180519, diluted 1:1000) and the proteasome subunit PSMA7 (PA5-21379, diluted 1:2000) as a loading control. Primary antibodies were incubated overnight at 4 °C. For detection we used a secondary goat-anti-rabbit antibody coupled to horseradish peroxidase (CAS 31466) and developed the membrane with Pierce ECL reagent (CAS 32106).

### Electron microscopy

#### ALYREF_55-182_–EJC–RNA structure

##### Negative stain electron microscopy of ALYREF_55-182_–EJC–RNA

ALYREF_55-182_–EJC–RNA was reconstituted on a 15 nt ssRNA as described above. After maltose elution, the complexes were separated and crosslinked by GraFix^73^ using a 15-40 % sucrose gradient supplemented with 0-0.07 % glutaraldehyde gradient spun at 50,000 rpm for 16 hrs in a SW60 Ti rotor. 200 μl fractions were collected and quenched for 15 min using a final concentration of 50 mM lysine. We separately pooled fractions corresponding to a light and a heavy species and buffer exchanged them into a buffer containing 25 mM HEPES, pH 7.9, 50 mM NaCl, 2 mM MgCl_2_ and 1 mM TCEP in a spin concentrator. Light and heavy fractions were analyzed by negative stain EM. For this, 4 μL of sample were applied to freshly glow-discharged copper grids coated with a 5 nm thick carbon film and incubated for 1 min. The grids were blotted, washed two times with 4 μL distilled water and stained for 1 min with 4 μl of 2 % (w/v) uranyl-acetate solution and blotted dry. We collected 85 micrographs of the light and 450 of the heavy fractions using SerialEM^74^ on a FEI Tecnai G2 20 transmission electron microscope with a (Eagle 4 k HS CCD camera) operated at 200 keV at a nominal magnification of 50,000x (2.21 Å pixel–1) and a target defocus range of –1 μm to –1.5 μm. 52,503 particles from the light fraction and 58,065 from the heavy were picked in WARP 1.0.9^68^. Light fraction particles were extracted in WARP 1.0.9 with a box size of 132 pixel, while heavy fraction particles were extracted with a 168 pixel box size in RELION 3.1^66^. Both data sets were imported in cryoSPARC^64^ for 2D classification.

##### Cryo-EM of the ALYREF_55-182_–EJC–RNA complex

Sample was prepared as described for negative stain analysis, and crosslinked gradient fractions corresponding to the hexamer were used for cryo-EM grid preparation. Buffer-exchanged sample was concentrated to 1.7 mg/mL and applied to Quantifoil Cu 200 3.5/1 grids and blotted at 4 °C and 75 % humidity and plunged into liquid ethane using a Leica EM GP. Data was collected on a Titan G4 with a Falcon 4 detector in counting mode at a nominal pixel size of 0.945 Å/px, a dose rate of 5.43 e^-^/px/sec and a total dose of 40 e^-^/Å^2^ in EER format. We used a 50 μm C2 aperture and no objective aperture. The energy filter was set to a slid width of 5eV. Data was collected using the Thermo Fischer EPU software, with autofocus routines being performed after 15 μm of stage movement. We acquired 24 movies per hole and a total of 7891 movies. Movies were motion- and CTF-corrected in cryoSPARC live^64^ and particles were picked in Warp^68^. We extracted 2,139,936 particles in a 320 px box that was fourier cropped to 128 pixels and performed *ab-initio reconstruction* with a subset of 100,000 particles. The obtained classes were low-pass filtered to 20 Å and used as templates for heterogenous refinement of the entire dataset. The best class contained 80% of particles which were re-extracted without furrier cropping and were subjected to two more rounds of heterogenous refinement (reference maps were low-pass filtered to 100 Å in the last round). The final particle set contained 1,564,602 particles and was refined to 2. 41Å resolution, using D3 symmetry. Refinement without applying symmetry (C1), yielded an indistinguishable reconstruction at slightly lower resolution (2.7Å)

##### Model building and refinement

We fitted the crystal structures of the EJC (PDB 2J0S)^23^ and the ALYREF RRM (PDB 3ULH) into our density and manually adjusted the model using COOT^75,76^. The model was refined using *ISOLDE*^77^ and in *phenix*^78,79^ using the phenix.real_space_refine routine with secondary structure and rotamer restraints. Figures were made with UCSF Chimera X^80,81^ and PyMol (https://www.pymol.org).

##### Sample preparation of TREX–EJC–RNA

To reconstitute the 10x-His-MBP-3C-ALYREF_N_–EJC–RNA complex, 10x-His-MBP-3C-ALYREF_N_ was mixed with a two-fold molar excess of 6x-His-3C-EIF4A3 and Y14 (66-154)-MAGOH dimer and a four-fold molar excess of the 50 nucleotide AdML-derived RNA^33^ in binding buffer (25 mM HEPES, pH 7.9, 50 mM NaCl, 5 mM MgCl_2_, 10 % (v/v) Glycerol, 1 mM TCEP, 1 mM AMP-PNP) and incubated overnight under rotation at 4 °C. The complex was then immobilized on HighFlow Amylose resin (NEB) equilibrated with binding buffer. 6x-His-TwinSTREPII-3C-UAP56 was incubated for 15 min at RT in buffer U (20 mM HEPES, pH 7.9, 60 mM NaCl, 5 mM MgCl_2_, 1 mM TCEP, 0.05 mM ATPγS) supplemented with PreScission protease. Then, the UAP56-3C protease mixture was added to the immobilized 10x-His-MBP-3C-ALYREF_N_–EJC–RNAand incubated at RT for 30min. Equimolar amounts of THO complex were added to the sample and further incubated for 30 min at RT and additional 30 min at 4 °C. The amylose resin was sedimented by centrifugation and the eluted TREX–EJC–RNA assembly was collected from the beads. 200 μl of TREX–EJC–RNA eluate was loaded onto a step gradient designed to separate THO–UAP56 from TREX–EJC complexes, this contained the following layers (listed bottom to top): 1.4 mL layer of buffer U supplemented with 40 % sucrose and 0.02 % glutaraldehyde (GA), 1.8 mL of buffer U supplemented with 20 % sucrose + 0.03 % GA, 0.8 mL of buffer U supplemented with 20 % sucrose without GA. The sample was centrifuged for 16 hrs at 20,000 rpm in a SW60 Ti rotor (Beckman coulter). We collected fractions and quenched the crosslinking reaction for 15 min using a final concentration of 50 mM lysine. Peak fractions were pooled, and buffer exchanged into 25 mM HEPES, pH 7.9, 50 mM NaCl, 2 mM MgCl_2_ and 1 mM TCEP in a spin concentrator.

We applied 4 μL of concentrated and crosslinked TREX–EJC–RNA complexes to glow-discharged CuR2/1 200 mesh holey carbon grids (Quantifoil). Grids were blotted at 4 °C and 75 % humidity and plunged into liquid ethane using a Leica EM GP.

##### Cryo-EM data acquisition of TREX–EJC–RNA

Data was collected at the electron bio-imaging center (eBIC) at the Diamond Light source (UK) on a Themo Fischer Titan Krios (instrument Krios IV) operated at 300 kV using a Gatan K3 direct electron detector operated in super-resolution mode and a BioQuantum post-column energy filter set to a slit width of 20 eV. A 70 μm C2 aperture and a 100 μm objective aperture were inserted. Data was collected at a pixel size of 1.06 Å/pixels (0.53 Å/pixels in super-resolution mode), a dose-rate of 14.949 e^-^/px/sec and a total dose of 63.6 e^-^ fractionated over 40 frames. The defocus range was set from -0.5 to -2 μm. Data was collected using the Thermo Fischer EPU software, and we acquired initially 8 images per hole, but changed during the session to 7 images per hole. We acquired a total of 12,938 micrographs.

### Data Processing of TREX–EJC–RNA

#### Pre-processing

Data was pre-processed using *Warp* v1.09^68^. Super-resolution movies were binned to 1.06 Å/px and contrast-transfer function (CTF) parameters were estimated with a spatial resolution of 3 by 3 and a fitting range from 30 Å to 5 Å. Motion correction was performed with a spatial resolution of 4 by 3. We picked 1,050,740 particles in *Warp* using a custom BoxNet model and extracted them in *RELION*^67^. Initially, we extracted particles in a 678 Å pixel box for 2D classification of the entire TREX–EJC density, but later re-extracted particles with a smaller box size of 340 Å and a binning factor of 1.25 to focus the classification on the ALYREF–EJC–RNA density.

#### Particle classification and refinement

Extracted particles were imported in cryoSPARC^64^. We used a cryo-EM density of MBP-ALYREF_55-182_–EJC complex previously obtained from an ab initio reconstruction from a dataset collected on a Glacios TEM microscope as a reference volume for a first round of heterogenous classification. In addition, we also performed ab initio reconstruction from the Krios dataset, yielding 3 classes. We then performed a second round of heterogenous classification with all particles in cryoSPARC, using the following maps as references: 1) the initial reference obtained from the glacios dataset, 2) one map obtained from the first round of heterogenous refinement, and 3) and 4) two maps obtained from *ab initio* reconstruction of the Krios dataset. The best class of that heterogenous classification corresponded to 36.5 % of the dataset and was used for further refinements with D3 symmetry and an ALYREF_N_–EJC–RNA mask. These comprised: a homogenous refinement, a CTF local refinement, a homogenous refinement, and a final non-uniform refinement, which yielded a density resolving at 3.0 Å with a B-factor of –190 Å^2^. Local resolution was estimated in cryoSPARC^64^. To confirm that the D3 symmetry operation did not introduce artifacts, we also generated a reference-free *ab-initio* model from the particles and refined the structure without imposing symmetry to 3.4 Å. Symmetrized and non-symmetrized maps were identical, apart from the slightly lower resolution in the latter.

### Electron microscopy of endogenous TREX–mRNPs

#### Negative stain analysis of native and nuclease digested TREX–mRNPs

##### Sample preparation

For native TREX–mRNPs, GFP-pulldown and elution was performed from NE from 24 L of K562 cells, and GFP pulldown was performed as described in *‘TREX–mRNP sedimentation coefficient determination*’. The eluted sample was crosslinked using GraFix^73^. The gradient was designed to concentrate particles in a narrow fraction and contained the following layers (listed bottom to top) 2 mL of binding buffer supplemented with 50% sucrose + 0.05% GA, 0.7 mL of binding buffer supplemented with 20% sucrose + 0.03% GA, 0.7 mL of binding buffer supplemented with 20% sucrose + 0.02% GA, and 600 μL of binding buffer supplemented with 15% sucrose without GA. 600 μl of eluted TREX–mRNPs were layered on top of the gradient and the sample was centrifuged for 16 hrs at 4 °C and 15,000 rpm in a SW60 Ti rotor. Gradients were fractionated in 200 μL fractions and quenched with 50 mM lysine. The peak fractions 10-12 (corresponding to the boundary between 20% sucrose and 50% sucrose) were pooled and buffer exchanged into 100 mM KCl, 25 mM HEPES, pH 7.9 and 1 mM TCEP in a spin concentrator. For nuclease digested TREX–mRNPs, sample was prepared in the same way, except that the peak was observed at fractions 7-11, which were pooled for EM analysis.

##### Negative staining and data analysis

Copper grids were coated with a ~5 nm homemade carbon film and glow-discharged. 4 μL sample was applied to the grid and incubated for 1 min. Grids were blotted and washed four times with 4 μL distilled water, stained for 1 min in 4 μL 2% (w/v) uranyl-acetate solution and blotted until dry. We collected 3,681 micrographs for the undigested TREX–mRNP sample (2,684 of benzonase treated sample) using *SerialEM*^74^ on a FEI Tecnai G^2^ 20 transmission electron microscope (Eagle 4 k HS CCD camera) operated at 200 keV at a nominal magnification of 50,000x (2.21 Å/px) and a defocus range of –1 μm to –1.5 μm. Particles were picked (87,921 and 131,140 for untreated and Benzonase treated, respectively) and extracted with a 400 pixel box size using *Warp* 1.09^68^ and transferred to RELION 3.1^67^ for 3D classification using a 40 Å low-pass filtered map of the human THO–UAP56 complex as a reference. Particles were classified into 4 classes, and the most featured class (12,964 and 39,104 particles, respectively) was selected and subjected to 2D classification.

##### Cryo-EM data acquisition endogenous TREX–mRNPs

Data was collected in two sessions at IST Austria on a Thermo Fischer Titan Krios G3i operated at 300 keV, equipped with Gatan K3 direct electron detector operated in counting mode and a BioQuantum post-column energy filter set to a slit width of 10 eV. A 50 μm C2 aperture was inserted, and the objective aperture was retracted. Both datasets were collected at a pixel size of 0.86 Å/px, a total dose of 60 e^-^ fractionated over 40 frames and a defocus range of -0.5 to –2 μm using SerialEM ^74^ For dataset 1, the dose rate was 13.37 e^-^/px/sec, and for dataset 2 the dose rate was 21.68 e^-^/px/sec. We collected 3 images per hole and 3 holes per stage position (totaling 9 images per stage position), with autofocus routines and drift measurements performed at every stage position. Dataset 1 and 2 comprised 11,756 and 10,182 micrographs respectively.

### Data processing of endogenous TREX–mRNPs

#### Pre-processing

Data was pre-processed using *Warp* v1.09^68^. CTF parameters were estimated with a spatial resolution of 6 by 4 and a fitting range from 25 Å to 3 Å. Motioncorrection was performed with a spatial resolution of 6 by 4. We picked 840,469 particles in total from both datasets in Warp using a custom BoxNet model and extracted them in *RELION*^67^ using a box size of 932 Å. For initial classification, particles were binned to 3.64 Å/px.

#### Particle classification and refinement

The cryo-EM data was classified in RELION 3.1 and cryoSPARC as described in Extended Data Fig. 5a. Briefly, the initial reference map for processing data sets 1 and 2 was obtained from the first 117,138 particles. These particles were subjected to 2D classification and then 3D classification in RELION 3.1 using 4 classes and the published THO–UAP56 map filtered to 60 Å as the 3D reference^28^. Class 3 was used as the reference map for processing the full set of particles. After two (dataset 1) or three (dataset 2) rounds of unmasked 3D classification in RELION 3.1, we separately extracted the two asymmetric dimers within each TREX tetramer unit with 1.41 Å/px (Extended Data Fig. 5d). This yielded a combined set of 415,848 dimer units from 21,938 micrographs (Extended Data Fig. 5a), which were then further classified using two rounds of heterogenous classification in cryoSPARC^64^ with three classes and a soft-edged mask surrounding TREX. We selected the class 3 particles in each round and refined these to 8.1 Å using the same TREX mask. We subsequently applied masks either around TREX monomers 1A and 2B (map A) or monomer 2B (map B) for individual focused 3D refinements (Extended Data Fig. 5b, c, d). Map A was refined at a nominal resolution of 3.9 Å and with a B-factor of –123 Å^2^, and confirms high-resolution structure features of THOC1, THOC2, THOC5, THOC6, and THOC7^28^. Map B was obtained after one additional round of 3D classification in cryoSPARC, using 10 classes and a mask on monomer B, yielding a major class with 61,269 particles that showed improved density for the N-terminal UAP56 RecA lobe and ALYREF UBM in monomer B. Local refinement yielded a map at a nominal resolution of 5.5 Å and with a B-factor of –202 Å^2^ and revealed additional low-resolution densities that belonged to UAP56, ALYREF, and mRNA. To resolve monomer 1A (map C), we performed 3D variability analysis and a subsequent focused 3D refinement of the combined particles from classes 3 and 6. This yielded map C at a nominal resolution of 7.8 Å and with a B-factor of –150 Å^2^ and showed density for the UAP56 RecA2 lobe. Local resolution was determined in cryoSPARC.

#### Model building

To prepare the TREX–mRNA complex model, we aligned maps A-C on their overlapping regions to generate a composite density, into which we rigid-body fitted the updated THO–UAP56 complex structure (see ‘Cryo-EM data processing of the recombinant THO–UAP56 complex’) in COOT^75,76^ (Extended Data Fig. 5e, f). We generated a UAP56–RNA–ALYREF_C_-UBM homology model based on the yeast crystal structure of Sub2–RNA–Yra1^41^ using MODELLER^82^, and then fitted only the UAP56 RecA1 lobe–RNA–ALYREFC-UBM into the density of map B using COOT^75,76^. We then replaced the ALYREF C-UBM helix with that from an AlphaFold model, obtained from a human UAP56–ALYREF C-UBM prediction, which better matched the density. The density surrounding the UAP56 RecA1 lobe has a local resolution of ~10 Å and showed an unambiguous rigid-body fit to the homology model (Extended Data Fig. 5g). This model shows a similar UAP56 RecA1 lobe–THOC2 interface as observed in yeast THO–Sub2 cryo-EM structures^5,31^. Notably, the ALYREF UBM helix (either N-UBM or C-UBM) and putative mRNA, bound to UAP56, are the only two contacts that connect the two-megadalton TREX complex to mRNPs.

### Cryo-EM data re-processing of the recombinant THO–UAP56 complex

To obtain high-resolution densities for the THO–UAP56 regions shared between monomer A and B, we re-processed the human THO–UAP56 cryo-EM data set^28^. Previously we had obtained monomer A and B densities at 4.6 Å and 4.7 Å respectively, precluding the assignment of protein or sidechain identity in the THOC2 C-terminal regions, which are identical in each monomer. First, we increased cryo-EM single particle numbers, by separately extracting the two asymmetric dimers within the high-quality THO–UAP56 tetramer units^28^. This yielded 314,583 dimer units from 26,303 micrographs (Extended Data Fig. 6a). Heterogenous classification was carried out in cryoSPARC^64^ with two classes and a soft-edged mask surrounding monomer A. We selected class 2 particles for their excellent density quality, whose subsequent processing resulted in maps D and E (Extended Data Fig. 6a-e). 3D refinement of class 2 particles with a mask surrounding THOC2, -3 and UAP56 yielded an overall resolution of 3.45 Å with a B-factor of 146 Å^2^(map D). We used another soft-edged mask to better resolve the THOC1, -2, -5, -7 N-terminal regions, yielding a 3D refinement from the same particles to 3.9 Å and a B-factor of 171 Å^2^(map E). Local resolution was determined in RELION 3.1^67^.

#### Model building

To generate the THO–UAP56 monomer A model, we aligned maps D and E via their overlapping regions to make a composite density (Extended Data Fig. 6d). Using COOT^75,76^ we then started from our previous backbone model of monomer A^28^ to build a near complete atomic model of THOC2 and THOC3 by adding missing loops, adjusting the amino acid register, and assigning sidechains. In our previous THO–UAP56 monomer A 4.6 Å density, we tentatively assigned a density at the THOC2 C-terminus as the THOC1 C-terminus. Using the new 3.45 Å map D of the same regions, we could now confirm this and build THOC1 residues 418-528 into unambiguous density in COOT^75,76^. An AlphaFold model of THOC1 residues 458-528 guided manual THOC1 modeling in COOT^75,76^. Using the 3.9 Å map E, we further extended our THO–UAP56 model to include poly-alanine models of the THOC2 ‘anchor’, and THOC5 and THOC7 N-terminal regions. We refined the coordinate model and B-factors using the phenix.real_space_refine routine with secondary structure and rotamer restraints^78,79^ (Table S1). The final THO–UAP56 complex model was obtained in COOT^75,76^ by replacing the former THO–UAP56 regions with the ones newly built here (Extended Data Fig. 6f).

### Cryo-electron tomography

#### Sample preparation

Cryo-electron tomograms were acquired on samples prepared identically and on the same day as samples for endogenous TREX–mRNP single particle analysis measurements.

#### Data acquisition

Tomograms were acquired on a Thermo Fischer Titan Krios G3i operated at 300 keV, equipped with Gatan K3 direct electron detector operated in counting mode and a BioQuantum post-column energy filter set to a slit width of 10 eV. A 50 μm C2 aperture and a 100 μm objective aperture were inserted. Data was acquired with *SerialEM*^74^ at a nominal magnification of 64,000x, resulting in a pixel size at the sample level of 1.38 Å/px. The dose rate was 18.3 electrons per pixel per second. Tilt series were collected using the dose-symmetric tilt scheme^83^. The tilt range was set to - 60° to +60° with a tilt increment of 2°. The total applied dose per tilt series was 120 e^-^/Å^2^, resulting in a dose of 1.97 e^-^/Å^2^/tilt movie. Tilt movies were fractionated over 6 frames (0.32 e^-^ / frame). Defocus targets for tilt series were set to -1.5 to -4 μm. A total of 120 tomograms were acquired.

### Data processing

#### Pre-processing

Tilt movies were motion- and CTF-corrected in *Warp* v1.09^68^, using models without spatial resolution. Next, tilt series stacks were exported for tilt series alignment in *etomo*^69^. Tilt series were aligned in batch using patch tracking without fiducials with the following parameters: data was binned 4x (resulting in a pixel size of 5.52 Å/px), and patch tracking was performed using tiles of 400x500 pixels and 0.8x fractional overlap between tiles. The average residual mean error after alignment was 1.45±0.47 Å. Of the 120 tilt series that were acquired, eleven could not be aligned and were discarded for downstream processing.

#### Tomogram reconstruction

Aligned tilt stacks were imported in *Warp*, and the tomogram dimensions specified to be 4,100x5,760x1,700 px (for the un-binned 1.38 Å/px pixel size). Tomogram CTF values were estimated in Warp (using aligned and averaged tilt movies, a CTF window size of 768 px, a data window fitting between 40 Å and 8 Å, and a defocus search range of 1 Å-8 Å). Defocus handedness was determined using *Warp’s* in-built tilt-handedness routine. Tomograms were reconstructed in *Warp* with a pixel size of 10 Å/px. We denoised each tomogram using *Warp’s* Noise2Map program, taking reconstructed tomograms from only odd or even tilts as input for the independent half-sets. Denoising was performed with 10,000 iterations. Denoising dramatically improved interpretability of tomograms and allowed us to visually identify TREX density in individual particles in many cases.

#### Template matching and particle classification

Template matching was performed using a 40 Å-lowpass-filtered reference map obtained from our single particle analysis. Template matching was performed in *Warp*, using a pixel size of 10 Å/pixel, 30° angular intervals, and cutoff for a minimal inter-particle distance of 150 Å between adjacent coordinates. These settings resulted in a total of 242,237 picked coordinates. Subtomograms of these coordinates were reconstructed with *Warp* with a pixel size of 5.0 Å/px and a box size of 180 px (900 Å).

Subtomograms were aligned in RELION 3.1^66,67^ using the same reference that was used for template matching and classified into four classes. Only one of these classes produced density that resemble TREX bound to an mRNP density (STA map 1, class 4, see Extended Data Fig. 9a). Particles belonging to this class were back-transformed into the original positions in their respective tomograms (see section ‘Visualization’) and their fit to the denoised density was assessed. Many particles showed poor fits, prompting us to further filter the particle set to obtain a high confidence set for downstream analysis. For this, we performed an independent template matching run, this time using the density of STA map 1 after denoising with *Noise2Map* as a reference, and our denoised tomograms as search targets. This approach yielded 59,275 coordinates, which were extracted (from the raw tomograms rather than the denoised tomograms which were used only for template matching), aligned and classified. The best class of this dataset contained 9,635 particles (STA map 2). To further increase stringency, we then calculated the overlap of the particles belonging to STA map 1 and STA map 2, keeping only those particles that were independently identified as belonging to a “good” class in both particle picking and processing strategies. This set contained 5,445 particles and was used to generate a reference free volume using the RELION *3D initial model* algorithm. The generated volume was used to further classify the merged particles of STA map1 and STA map 2 using three rounds of 3D classification (see Extended Data Fig 8a). After the first round, duplicated particles were removed (distance cutoff 100 Å). In the last round of 3D classification, less then 0.1 % of particles were classified into “junk” classes, indicating convergence. This final particle set contained 10,105 particles and was refined in RELION without symmetry. Postprocessing with a mask encompassing the entire C2-symmetric TREX complex yielded an overall resolution estimate of 17 Å, whereas tighter masks on the “scaffold” made up by THOC5, -6, and -7 yielded an estimate of 15 Å and a tight mask on monomer B (THOC1, -2, and -3) yielded an estimate of 13 Å. Please note that this processing strategy aimed to keep only the highest confidence TREX particles (minimizing false positives), but resulted in many TREX particles that were missed, as was evident from inspecting denoised tomograms after overlaying the final particle set. Therefore, we choose not to make any statements about the average number of TREX molecules per mRNP, as this number would vastly underestimate the true value.

### Tomogram analysis

#### Measurement of TREX–mRNP volumes and sphericity

TREX–mRNP volumes were measured in denoised tomograms using the *measure blob* function in *ChimeraX*, which reports the volume enclosed within an iso-surface at a given threshold, as well as the dimensions of the 3 principal axes enclosing the volume. Sphericity was calculated by diving the length of the shortest axis by that of the longest axis. Only particles fully separated from neighboring particles were measured. Measurements were done at a relatively stringent threshold of 0.025, because at lower thresholds particles started touching each other due to the high particle density in our sample. Dimensions of native (no nuclease treatment) TREX–mRNPs were determined on micrographs of uranylacetate stained particles acquired on a FEI Tecnai T20 microscope using ImageJ^70^.

#### Visualization

To generate annotated tomograms with positions of TREX identified from our subtomogram averaging analysis, we used the program *peet*^84,85^. For this, we extracted particle poses and coordinates from RELION star files and converted them to *imod* ‘slicer angles’ using a combination of custom bash scripts, the RELION StarTool python package (C. Dienmann, https://github.com/cdienem/StarTool) and the programs RELION2MOTL and MOTL2Slicer^84,85^. To generate a map that shows the position of TREX in the original tomograms, we fitted the atomic model of TREX (this study) into the subtomogram average map, and simulated TREX density at a resolution of 20 Å using a pixel size of 10 Å, to match that of the reconstructed tomograms. This map was cloned into empty tomograms using the poses obtained from subtomogram averaging using the *imod* program *clonevolume*, generating for each tomogram a volume that shows the positions of TREX confidently identified in our high stringency particle set. We refer to this volume that shows the position of TREX as identified through the subtomogram averaging workflow as the ‘TREX positions map’ hereafter.

#### Extracting atomic models of TREX pairs

First, we fitted PDBs of TREX into our TREX positions maps. For this, we used the *fitmap* command in *UCSF ChimeraX* v1.3^80,81^ to generate 10,000 initial random placements of the PDB into each TREX positions map. Initial fits were refined, and all fits with correlations scores >0.93 were considered true-positives and a copy of the PDB at the identified position was saved. Results of this automated procedures were inspected, and any missed densities manually fitted. Next, we manually inspected 56 tomograms for the presence of particles where two copies of TREX bound to the same mRNP were identified in our high-stringency particle set. For this, denoised tomograms, TREX positions maps, and fitted PDBs of TREX were overlayed in *ChimeraX*. For each identified TREX pair, the fit of the TREX clone density to the denoised tomogram density was visually inspected, and obvious false positives were rejected. This procedure yielded 275 TREX pairs, and 11 instances of TREX ‘triples’ (three copies of TREX bound to the same mRNP), which were represented as pseudo-atomic models through the fitted PDBs. For visualization purposes, we next used ChimeraX to align all TREX pairs to a common reference, generating each of the two possible alignments given TREX’s C2 symmetry.

#### TREX–TREX distance and orientation measurements

We measured for each pair the TREX–TREX distance, using the centrally located residue THOC5-K516 as reference points for the measurement. To express relative orientations of two copies of TREX (TREXA and TREXB) bound to the same mRNP, we calculated rotation matrixes that align TREX_B_ with TREX_A_ using the ChimeraX *align* command. Given the C2 symmetry of TREX which results in two equivalent ways to achieve the same alignment, we first measured pairwise distance of symmetry related copies of THOC1 between the two TREX copies TREX_A_ and TREX_B_ (distances from THOC1^1^_TREXA_ to THOCP_TREXB_, THOC1^1^_TREXA_ to THOC1^2^_TREXB_, THOC1 2TREXA to THOC1^1^_TREXB_, THOC1 ^2^_TREXA_ to THOC1^2^_TREXB_) and then aligned the copies of THOC1 that where closest to each other. The obtained rotation matrices were converted to Euler angles (using the convention of intrinsic, right-handed rotations around the axis X, Y and Z) with the python package *eulerangles* (A. Burt, https://github.com/alisterburt/eulerangles). As a result, each of the 275 identified TREX pairs was annotated with their center-to-center distance and their relative orientation to another expressed in Euler angles.

#### Plotting of TREX–TREX orientations

In order to visualize the TREX–TREX orientation distribution in our dataset, we reduced our four-dimensional data (three angles and one distance measurement) to two dimensions using the t-Distributed Stochastic Neighbor Embedding (t-SNE)^50^ dimensionality reduction approach implemented in the *R* package *Rtsne*^86^. To generate the heatmaps of TREX–TREX vector angles (Extended Data Fig. 10c), TREX–TREX vectors (expressed in cartesian format in the rotation matrix obtained through the ChimeraX *align* command) were converted to spherical coordinates using s custom python script and the angles were plotted using *R*^87^ and *ggplot*^88^.

### Crosslinking mass spectrometry (crosslink MS)

For crosslinking MS analysis, we produced NE from a total of ~85 L K562 cells in four batches. Nuclease digestion was omitted for these samples. TREX–mRNPs were purified via a GFP-pulldown as described above, but Glycerol and Igepal were only included in the first three washes and omitted in subsequent steps. The beads were additionally washed three times and eluted with 3C protease as before. The eluted sample was split in three and sulfo-sulfosuccinimidyl 4,4'-azipentanoate (Sulfo-SDA, Thermo Fischer Scientific) was added to each at final concentrations of 1 mM, 2.5 mM and 4 mM, respectively. The reaction was allowed to occur for 30 minutes at room-temperature in the dark. The sample was then spread in a thin film in 6-well tissue-culture treated plates and placed on ice-cooled metal blocks underneath a UV lamp (UVP Blak-Ray B-100AP at 468, wavelength of 365 nm) for 40 min. The sample was then collected, quenched with 50 mM ammonium bicarbonate for 10 min, and precipitated by adding four volumes of -20 °C cold acetone and incubating for 60 min at -20°C. Precipitated material was pelleted by centrifugation at 15,000 g for 10 min, the pellet was briefly washed in cold acetone, pelleted again, and air-dried. The crosslinked samples were resuspended in 8 M urea, 100 mM ammonium bicarbonate and 0.1 % RapiGest SF (Waters). Proteins were digested with Lys-C (Pierce) at an enzyme-to-substrate ratio of 1:100 for 4 hrs at 22 °C. Peptides were reduced with 5 mM DTT and alkylated with 15 mM iodoacetamide. Following alkylation and after diluting the urea to 1.5 M with 100 mM ammonium bicarbonate solution, the peptides were further digested with trypsin (Pierce) at an enzyme-to-substrate ratio of 1:20 for 16 hrs at 22 °C.

Digested peptides were eluted from StageTips and split into two, for parallel crosslink enrichment by strong cation exchange chromatography (SCX) and size exclusion chromatography (peptideSEC), and were dried in a vacuum concentrator (Eppendorf, Germany). For SCX, eluted peptides were dissolved in mobile phase A (30 % acetonitrile (v/v), 10 mM KH_2_PO_4_, pH 3) prior to strong cation exchange chromatography (100 x 2.1 mm Poly Sulfoethyl A column; Poly LC, Colombia, MD, USA). The separation of the digest used a non-linear gradient into mobile phase B (30 % acetonitrile (v/v), 10 mM KH_2_PO_4_, pH 3, 1 M KCl) at a flow rate of 200 μl/min. Sixteen 1 min fractions in the high-salt range were collected and cleaned by StageTips, eluted and dried for subsequent LC-MS/MS analysis. For peptideSEC, peptides were fractionated on an ÄKTA Pure system (GE Healthcare) using a Superdex 30 Increase 3.2/300 (GE Healthcare) at a flow rate of 10 μl/min using 30% (v/v) acetonitrile and 0.1% (v/v) trifluoroacetic acid as mobile phase. Five 50 μl fractions were collected and dried for subsequent LC-MS/MS analysis.

Samples for analysis were resuspended in 0.1% v/v formic acid, 3.2 % v/v acetonitrile. LC-MS/MS analysis was conducted in duplicate for SEC fractions and SCX fractions, performed on an Orbitrap Fusion Lumos Tribrid mass spectrometer (Thermo Fisher Scientific, Germany) coupled on-line with an Ultimate 3000 RSLCnano system (Dionex, Thermo Fisher Scientific, Germany), running Xcalibur 4.1. The sample was separated and ionized by a 50 cm EASY-Spray column (Thermo Fisher Scientific). Mobile phase A consisted of 0.1 % (v/v) formic acid and mobile phase B of 80 % v/v acetonitrile with 0.1 % v/v formic acid. The flow-rate was 0.3 μl/min using gradients optimized for each chromatographic fraction from offline fractionation ranging from 2% mobile phase B to 45% mobile phase B over 120 min, followed by a linear increase to 55 % and 95 % mobile phase B in 2.5 min, respectively. The MS data were acquired in data-dependent mode with a 2.5 sec cycle time. For every cycle, the full scan mass spectrum was recorded in the Orbitrap at a resolution of 120,000 in the range of 400 to 1,600 m/z. Ions with a precursor charge state between 3+ and 7+ were isolated and fragmented. Fragmentation was performed by stepped higher-energy collisional dissociation (HCD) using 26, 28 and 30 %. The fragmentation spectra were then recorded in the Orbitrap with a resolution of 60,000. Dynamic exclusion was enabled with single repeat count and 60-second exclusion duration.

A RecA1ibration of the precursor m/z was conducted based on high-confidence (<1% FDR) linear peptide identifications. To identify crosslinked peptides the RecA1ibrated peak lists were searched against the sequences and the reversed sequences (as decoys) of crosslinked peptides using the Xi software suite (version 1.7.6.4)^89^ (https://github.com/Rappsilber-Laboratory/XiSearch). The following parameters were applied for the search: MS1 accuracy = 2 ppm; MS2 accuracy = 5 ppm; enzyme = trypsin allowing up to 3 missed cleavages and 2 missing monoisotopic peaks; crosslinker = SDA with an assumed NHS-ester reaction specificity for lysine protein N termini; fixed modifications = carbamidomethylation on cysteine; variable modifications = acetylation on lysine and protein N-termini, oxidation on methionine, hydrolysed SDA on lysines and protein N-termini. MS-cleavage of SDA crosslinks is considered during search. Prior to FDR estimation the matches were filtered to those having at least two fragments matched with a non-cleaved SDA. These candidates were then filtered to 2% FDR on PPI-level using XiFDR (version 2.1.5.2)^90^.

### Estimation of UBM saturation on mRNPs

We first calculated local UBM concentration within a sphere with a diameter of 450 Å, corresponding to the median mRNP as measured from negative stain micrographs (Extended Data Fig 4f). The median human mRNA contains 8 introns, corresponding to 8 ALYREF molecules, assuming stochiometric binding to the EJC. Given that the N-and C-terminal UBMs show near-identical affinities to UAP56^31^, we treated N- and C-terminal UBMs as equivalent, resulting in 16 UBM per mRNP^91^ or 560 μM.

To estimate concentrations of UAP56 and THO complex in the nucleoplasm, we converted published protein copy numbers of UAP56 from mouse embryonic fibroblasts^52^ into molar concentrations assuming a spherical nucleus with 15 μm in diameter, yielding a nuclear UAP56 concentration of 3.2 μM.

Given a K_D_ of 2.9 μM for the UBM-ALYREF interaction^31^, and a local UBM concentration of 560 μM, this results in 99.5% saturation of binding (calculated using the online tool https://share.streamlit.io/wjiang/protein-ligand-binding), suggesting that for an average fully spliced mRNA the UBM-UAP56 interaction is sufficient to recruit UAP56. Our *in vitro* experiments indicate that THO–UAP56 has a higher affinity than UAP56-ALYREF (Extended Data Fig. 7), and we therefore assume that all THO is bound to UAP56 at steady state. Considering the tetrameric architecture of THO–UAP56, this translates to ~3 TREX copies per mRNP.

### GFP-protein accessibility probing of endogenous mRNP complexes using an anti-GFP nanobody

For the GFP-tag accessibility assays in Extended Data Fig. 10, we generated cytoplasmic and nuclear extract from K562 cells carrying N-terminal homozygous GFP-tags on either EIF4A3 or THOC5. For experiments with cytoplasmic extract, the extract was concentrated 10-fold in a 100 kDa molecular weight-cutoff due to the lower abundance of EIF4A3 in the cytoplasm compared to the nucleoplasm. The extracts were prepared as for TREX–mRNP purification using a mild nuclease digestion, extracts were added 1 μg Benzonase per mL NE in absence of Mg^2+^. 150 μL of each NE was then incubated with 0.5 μL of 5 μM GFP-nanobody for 1 hrs at 4 °C (FluoTag-Q anti-GFP, CAS N0301-AF647-L). Extracts were then diluted two-fold with a buffer containing 100 mM KCl, 20 mM HEPES, pH 7.9 and 2 mM MgCl_2_ and immediately applied to 15-40 % sucrose gradients and centrifuged for 16 hrs at 15,000 rpm and 4 °C. Gradients were fractionated in 200 μl fractions. 40 μL of every other fraction were mixed with 10 μL of 5x SDS-PAGE loading dye and 40 μl were immediately loaded (without boiling) on NuPage 4-12 % BisTris gels. Gels were run at 125 V for 50 minutes. Under these conditions, the GFP-nanobody remained bound to GFP-tagged proteins during gel electrophoresis and GFP fluorescence was preserved. Gels were imaged on a laser scanner (Sapphire scanner, Azure BioSystems) with a pixel size of 100 μm and fluorescence in the GFP channel and AF647 channel was measured. GFP-fluorescence and AF647-GFP-nanobody fluorescence were quantified in ImageJ^92^, and background-corrected intensity values were normalized in GraphPad Prism. Quantifications were performed from three technical replicates.

## Extended Data

**Extended Data Table 1:** Cryo-EM data collection and refinement statistics Cryo-EM data collection and refinement statistics of the ALYREF–EJC–RNA (ALYREF55-182–EJC–RNA and TREX–EJC–RNA), TREX–mRNP, and THO–UAP56 structures. Coordinate model statistics are only indicated once per deposited structure.

	ALYREFss-1s2-EJC-RNA (EMDB-14803) (PDB 7ZN J)	TREX-EJC­RNA (EMDB-16633)	TREX-mRNA Map A (EMDB- 14805) (PDB 7ZNK)	TREX-mRNA Map B (EMDB- 14806)	TREX-mRNA Map C (EMDB- 14807)	THO-UAP56 Map D (EMDB- 14808 (PDB 7ZNL)	THO-UAP56 Map E (EMDB- 14809)
Data collection and processing							
Magnification	130,000	81,000	105,000	105,000	105,000	105,000	105,000
Voltage (kV)	300	300	300	300	300	300	300
Electron exposure (e-/Å^2^)	40	64	60	60	60	50	50
Defocus range (μm)	-0.6 to -2.5	-0.5 to -2.0	-0.3 to -2.5	-0.3 to -2.5	-0.3 to -2.5	-0.4 to -3.7	-0.4 to -3.7
Pixel size (Å)	0.945	1.06	0.86	0.86	0.86	0.86	0.86
Symmetry imposed	D3	D3	Cl	Cl	Cl	Cl	Cl
Initial particle images (no.)	2,139,936	1,050,740	840,469 (tetramers)	840,469 (tetramers)	840,469 (tetramers)	3,140,530 (tetramers)	3,140,530 (tetramers)
Final particle images (no.)	1,564,602	383,510	182,534 (dimers)	61,269 (dimers)	29,591(dimers)	246,457 (dimers)	246,457 (dimers)
Map resolution (Å)	2.4	3.0	3.9	5.5	7.5	3.45	3.9
FSC threshold		0					
	0.143	0.143	0.143	0.143	0.143	0.143	0.143
Map resolution range (Å)	2.4-2.8	2.8-3.2	3.7-4.1	4.5-10.0	7.0-12	3.3-4.9	3.7-5.0
Refinement							
Initial model used (PDB code)		2JOS	7APK			7APK	
Model resolution (Å)	2.5	3.1	7.2	7.1	8.8	4.1	7.1
FSC threshold	0.5	0.5	0.5	0.5	0.5	0.5	0.5
Model resolution range (Å)							
Map sharpening B factor (Å^2^)	-100	-190	-134	-202	-913	-146	-171
Model composition							
Non-hydrogen atoms	35,088		88,301			84,829	
Protein residues	4,242		11,599			11,173	
Ligands	12		0			0	
*B* factors (Å^2^)							
Protein	16		Not refined			115	
RNA	30		Not refined				
Ligand	3						
R.m.s. deviations							
Bond lengths (Å)	0.003		0.006			0.004	
Bond angles(°)	0.770		1.098			0.720	
Validation							
MolProbity score	1.09		2.69			2.46	
Clashscore	3.02		9.82			7.64	
Poor rotamers (%)	0.86		8.7				
Ramachandran plot							
Favored (%)	99		93.3			93.1	
Allowed (%)	1		6.4			6.59	
Disallowed (%)	0		0.3			0.3	

## Supplementary Material

Supplementary Table 1

Supplementary Table 2

Supplementary Table 3

Supplementary Video 1

Supplementary Video 2

Supplementary Video 3

Supplementary Video 4

Supplementary Video 5

Supplementary Video 6

## Data Availability

Three-dimensional cryo-EM density maps of the ALYREF_55-182_–EJC–RNA complex, TREX–EJC–RNA complex, TREX–mRNA complex (composite map and maps A, B, C), THO–UAP56 complex (maps D, E) have been deposited in the Electron Microscopy Data Bank under the accession numbers EMD-14803, EMD-16633, EMD-14804, EMD-14805 EMD-14806, EMD-14807, and EMD-14808, and EMD-14809 respectively. The coordinate file of the ALYREF–EJC–RNA, TREX–mRNA, and THO–UAP56 complexes have been deposited in the Protein Data Bank under the accession numbers 7ZNJ, 7ZNK, and 7ZNL. Protein crosslinking data have been deposited in JPOST and ProteomeXchange with the accession codes JPST001488 and PXD031755, respectively.
